# Bi-2212/1T-TaS_2_ Van der Waals junctions: Interplay of proximity induced high-*T*_*c*_ superconductivity and CDW order

**DOI:** 10.1038/s41598-017-04645-1

**Published:** 2017-07-05

**Authors:** Ang J. Li, Xiaochen Zhu, G. R. Stewart, Arthur F. Hebard

**Affiliations:** Department of Physics, University of Florida, Gainesville, FL 32611 USA

## Abstract

Understanding the coexistence, competition and/or cooperation between superconductivity and charge density waves (CDWs) in the transition metal dichalcogenides (TMDs) is an elusive goal which, when realized, promises to reveal fundamental information on this important class of materials. Here, we use four-terminal current-voltage measurements to study the Van der Waals interface between freshly exfoliated flakes of the high-*T*_*c*_ superconductor, Bi-2212, and the CDW-dominated TMD layered material, 1T-TaS_2_. For highly transparent barriers, there is a pronounced Andreev reflection feature providing evidence for proximity-induced high-*T*_*c*_ superconductivity in 1T-TaS_2_ with a surprisingly large energy gap (~20 meV) equal to half that of intrinsic Bi-2212 (~40 meV). Our systematic study using conductance spectroscopy of junctions with different transparencies also reveals the presence of two separate boson modes, each associated with a “dip-hump” structure. We infer that the proximity-induced high-*T*_*c*_ superconductivity in the 1T-TaS_2_ is driven by coupling to the metastable metallic phase coexisting within the Mott commensurate CDW (CCDW) phase and associated with a concomitant change of the CCDW order parameter in the interfacial region.

## Introduction

In the past few decades, the study of interfaces between novel materials including metals, semiconductors, superconductors, topological insulators and layered materials harboring charge density waves (CDWs) has generated the emergence of unexpected phenomena. These discoveries require a new understanding of underlying mechanisms which in turn may well lead to promising new technologies. The proximity effect at the superconducting/normal (S/N) boundary posits a leakage of Cooper pairs into the normal metal accompanied by the appearance of superconductivity at the interface and extending into the metal^1–3^. Specifically, detailed study of S/N proximity junctions provides a unique probe of the pairing interaction of both conventional^1^ and unconventional^2^ superconductors. The reflection of an electron in N as a hole of opposite wave vector (Andreev reflections) and propagation of paired quasiparticles in S gives rise to phenomena including contact-dependent excess conductance, reduced energy gaps and lower transition temperatures on both sides of the interface. Van der Waals (VdW) interfaces advantageously reduce concerns about epitaxial matching across the interface and have already been studied using ultra-smooth cleavable superconductors such as: Bi-2212 in contact with the topological insulators, Bi_2_Se_3_ and Bi_2_Te_3_^4^, 2H-NbSe_2_ in contact with Bi_2_Se_3_^5^ and 2H-NbSe_2_ in contact with graphene^6^.

In this paper we are motivated by the question of how coexistence, competition or cooperation of superconductivity with various collective electronic states, in particular charge density wave ordered states, can be studied using the proximity effect. This question has been discussed in systems of reduced dimensionality which are prone to electronic instabilities, such as the *coexistence* of CDW order and superconductivity in 2H-NbSe_2_^7^ in contrast to the *competition* of CDW order and superconductivity in, for example, Yttrium cuprate^8^. The quasi-two dimensional layered transition-metal dichalcogenides (TMDs), many of which harbor CDW order, have served as model systems for the investigation of the interplay between CDWs and low *T*_*c*_ superconductivity where low-*T*_*c*_ superconductivity can be achieved by the application of pressure^9^ to pristine 1T-TaS_2_ or by partial substitution of Se for S^10^, Fe for Ta^11^ or electric-field gate controlled intercalation of Li^12^ and is always associated with the more conducting nearly commensurate CDW (NCCDW) phase and even the higher temperature incommensurate CDW (ICCDW) phase. Interestingly, in the pressure experiments on pristine 1T-TaS_2_, the low-*T*_*c*_ superconductivity (*T*_*c*_ ≈ 5 K) persists until the pressure is high enough to convert the CDW phase to a metal^9^.

The TMD, 1T-TaS_2_, is a particularly interesting CDW material for such studies because it exhibits a pronounced first order CDW transition with hysteretic resistance transitions in the 180–230 K temperature range. It is generally recognized that electron–electron as well as electron–phonon interactions in 1T-TaS_2_^13–16^ are responsible for the evolution of a nearly commensurate CDW (NCCDW) to a Mott commensurate CDW (CCDW) phase dominating at low temperatures. Two opposing arguments focus on where and how low *T*_*c*_ superconductivity forms in 1T-TaS_2_. The first argues that the superconductivity is formed within the metallic interdomain spaces separating the CCDW domains where tens of “star of David” clusters clump into rough hexagonal domains reproducing the Mott-CCDW phase locally^9, 10^. The second argues that the superconductivity, characterized by a shallow electron pocket at the Brillouin-zone center, is formed exactly within the clusters of stars^11^ in the NCCDW phase in real space. We note that low-*T*_*c*_ superconductivity at ambient pressure is only found in non-pristine (i.e., doped) 1T-TaS_2_ where the NCCDW phase dominates.

In this study, we find evidence for proximity-induced high-*T*_*c*_ superconductivity in the topmost layers of pristine 1T-TaS_2_ at the interface of Van der Waals bonded Bi-2212/1T-TaS_2_ junctions, where pristine Bi-2212 is the high-*T*_*c*_ superconductor Bi_2_Sr_2_CaCu_2_O_8+*δ*_ with a transition temperature *T*_*c*_ = 85 K and intrinsic energy gap Δ_0_ = 40 meV. Andreev reflection, marked by excess current and a wide zero-bias conductance peak, is observed at temperatures up to 80 K where the 1T-TaS_2_ is in the Mott-CCDW state. The proximity induced gap Δ_*a*_ in the 1T-TaS_2_ is found to be a surprisingly large 20 meV, thereby implying a strong coupling limit (2Δ_*sc*_/*k*_*B*_ *T*_*c*_ ~ 5.8) instead of the BCS weak coupling limit of 3.5. In addition to the induced gap in the 1T-TaS_2_, we also observe a depressed gap compared to the intrinsic gap Δ_0_ in the vicinity of the interface on the Bi-2212 side.

Our observation of a transparency-dependent superconducting proximity effect of Bi-2212/1T-TaS_2_ junctions strongly indicates that superconductivity is induced in a metallic phase of 1T-TaS_2_. The transparency is defined as a dimensionless normal conductance, ${\sigma }_{N}=\frac{1}{1+{Z}^{2}}$, where the parameter Z is extracted from theoretical fitting of conductance spectroscopy data using the extended BTK model described below. Such a finding is somewhat surprising since, in the absence of intimately contacting Bi-2212, pristine 1T-TaS_2_ is in an insulating CCDW state, and a proximity effect is only expected to occur in metallic systems. The puzzle here is that at the temperatures where Bi-2212 is superconducting, the stoichiometric pure 1T-TaS_2_ is in the nonmetallic Mott-CCDW state and a proximity effect is in fact observed thereby implying that the proximity of the Bi-2212 induces changes in the CDW order parameter to achieve a more metallic phase. Said in another way, the mutual interaction of the CDW and superconducting order parameters is such that the proximity-induced gap in the 1T-TaS_2_ can only appear if the CDW order parameter is changed in the interfacial region as is the superconducting gap associated with the Bi-2212. This interpretation is consistent with evidence for a metastable metallic phase, induced by voltage pulses, laser pulses or current excitations, residing within the Mott-CCDW phase^17–21^. This metastable state is ascribed to the reduction of onsite Coulomb interaction *U* and increase of Hubbard band width *W* via phase shifts of the CDW order parameter or interplay between the electron-electron and electron-phonon interactions in the topmost layers^17–21^.

The superconducting proximity effect, attributed to the leakage of Cooper pairs into a conducting metallic phase of the 1T-TaS_2_, is also confirmed by the good agreement of our *c*-axis conductance spectroscopy (*dI*/*dV* vs. *V*) with an extended BTK model for *d*–wave superconductors^22^. Additionally, our conductance measurements reveal the presence of two dip–hump structures which can be interpreted to reflect an inherent electron–phonon interaction in 1T-TaS_2_ that assists the formation of high-*T*_*c*_ superconductivity in the metastable metallic domains residing within the Mott-CCDW phase. This evidence of electron–phonon interaction assisted high-*T*_*c*_ superconductivity within the Mott CCDW phase of 1T-TaS_2_, presents a new paradigm for understanding the correlation of CDW order and high-*T*_*c*_ superconductivity in 1T-TaS_2_.

## Results

### Characteristics of Samples

Nearly optimally doped crystals of high-*T*_*c*_ cuprate Bi_2_Sr_2_Ca_1_Cu_2_O_8+*δ*_ (Bi-2212) and stoichiometically pure layered transition metal dichalcoginide (TMD) 1T-TaS_2_ crystals were used. The critical superconducting temperature *T*_*c*_ of Bi-2212 (Supplementary Fig. S.1(a)) and the CCDW-NCCDW phase transition temperatures on cooling/warming processes (Supplementary Fig. S.1(b)) were verified with AC transport measurements to be at 85 K and 180 K/230 K respectively.

**Figure 1:**
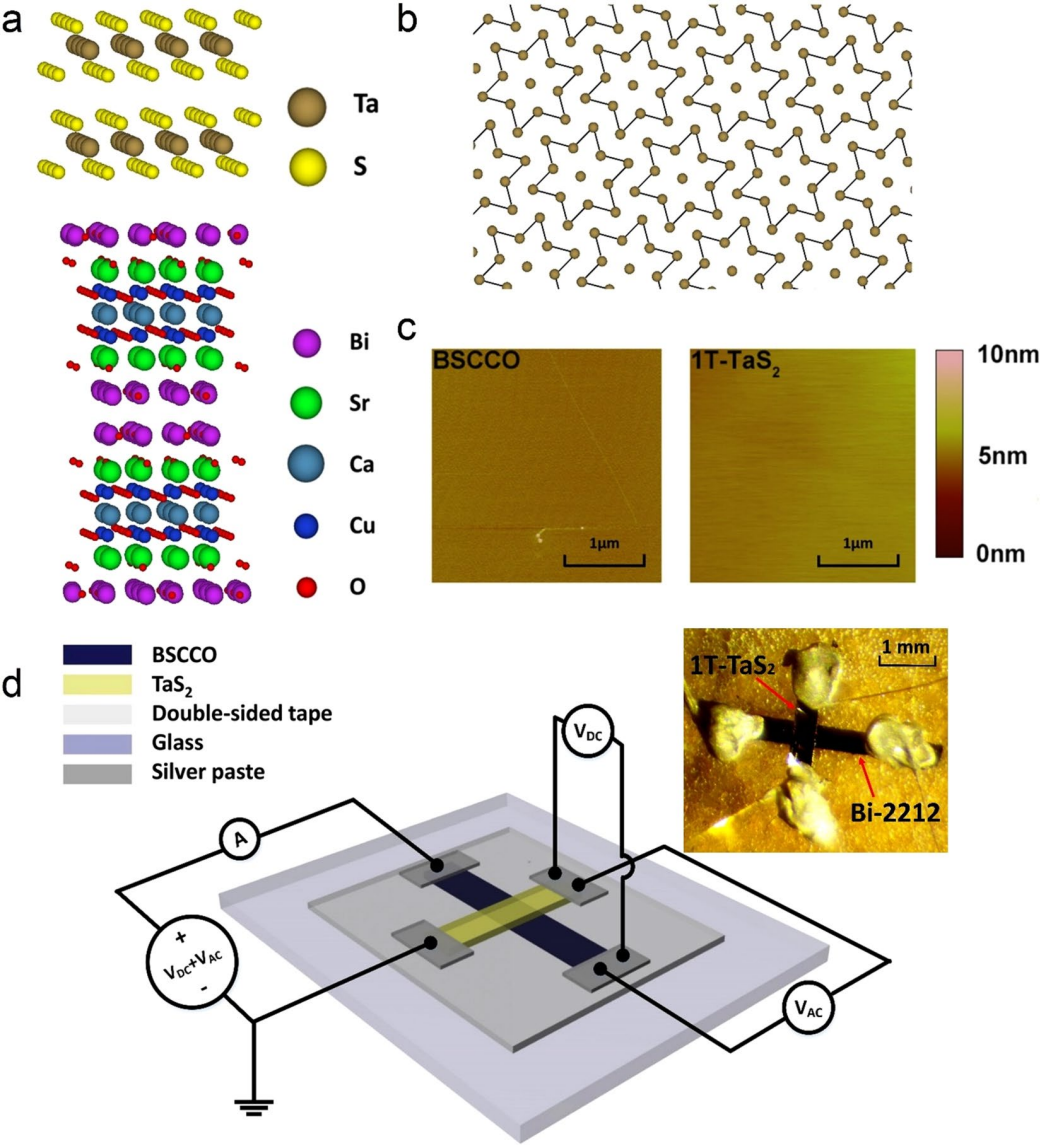
Sample characteristics and measurement set-up. (**a**) Crystal structure of 1T-TaS_2_ (upper schematic) and Bi-2212 (lower schematic) viewed along the direction parallel to the *ab*-plane. (**b**) Schematic of monolayer 1T-TaS_2_ along the *c*-axis in CCDW state. The interlocked clusters of Ta atoms (“star of David”) are sketched in dark yellow for the Ta atoms and the chemical bonds between Ta atoms, excluding the central one, are sketched in black. The S atoms are not shown. (**c**) Atomic force microscope (AFM) images of the cleaved surfaces for thin flakes of Bi-2212 (left) and 1T-TaS_2_ (right). The scale bar corresponds to 1 *μm*. The surface mean roughness of thin flake Bi-2212 and 1T-TaS_2_ are 1.55 Å and 1.52 Å respectively. (**d**) Schematic depicting experimental set-up for making four-terminal current-voltage and differential conductance vs voltage measurements using an AC + DC adder in conjunction with one DC voltmeter and two synchronized lock-in amplifiers, one for AC current and the other for AC voltage. Inset: photograph of Bi-2212/1T-TaS_2_ device. The scale bar corresponds to 1 mm.

The Bi-2212 and 1T-TaS_2_ crystals were mechanically exfoliated in a dry atmosphere as thin flakes with approximately rectangular shapes and nominal thicknesses of 0.5–2.0 *μ*m and 2.0–5.0 *μ*m for the Bi-2212 and 1T-TaS_2_ flakes respectively. The cleaved surfaces were clean and flat, with a mean roughness of 1.55 Å for Bi-2212 and 1.52 Å for 1T-TaS_2_ from AFM images as shown in Fig. 1(c). Two cleaved thin flakes were placed against each other and naturally bonded via Van der Waals forces^23^; high quality normal metal-superconductor (NS) or normal metal-insulator-superconductor (NIS) junctions were then formed. Four terminal tunnel junction configurations with perpendicularly oriented top and bottom electrodes shown in the Fig. 1(d) schematic have the advantage that contact resistances are eliminated and the active area common to both electrodes (0.2 mm^2^) can be accurately calculated^24, 25^. Advantageously the *c*-axis of both the bottom (Bi-2212) and top (1T-TaS_2_) electrodes are perpendicular to the substrate and thus colinear, thereby minimizing currents flowing along the *ab*-plane. Our *c*-axis conductance spectroscopy measurements were performed in a Quantum Design Physical Properties Measurement System (PPMS) at temperatures ranging from 2.5 K to 120 K. Using this set up we find the intrinsic Bi-2212 superconducting gap to be in the range 38 to 42 meV for both Bi-2212/1T-TaS_2_ junctions (see below) and Bi-2212/graphite (Supplementary Fig. S.2) junctions, in good agreement with previous point-contact tunneling studies on single crystal Bi-2212^26, 27^.

### Experimental measurements of Bi-2212/1T-TaS_2_ junctions

#### Highly transparent junctions

C-axis AC differential conductance spectroscopies and DC current-voltage (*I*–*V*) characteristics were measured on Bi-2212/1T-TaS_2_ junctions. To exemplify the superconducting proximity effect at the interface, junctions with high transparency^1, 3, 28, 29^ or low Z parameter described in the BTK model^29^ are needed. Under these conditions, Cooper pairs in the superconductor (S) can leak into the normal material (N) side resulting in a spatially dependent superconducting gap Δ(*x*) that extends across the interface along the *c*-axis and into the 1T-TaS_2_. Various gap features can be identified beginning with the intrinsic superconducting gap Δ_0_ = 40 mV deep in the superconductor which decreases to a depressed superconducting gap Δ_*p*_ = 28 mV at the S/N interface and then decreases to a proximity effect induced superconducting gap Δ_*a*_ = 20 mV on the N side of the interface which decreases in magnitude with increasing distance from the interface^1, 3^. Moreover, the different proximity region widths within which these gaps exist depend sensitively on the transparency of junctions^1, 3, 30–32^. In addition, different quasi-particle lifetimes or scattering rates at the vicinity of interface might affect the spectroscopic line shapes^33^.

**Figure 2:**
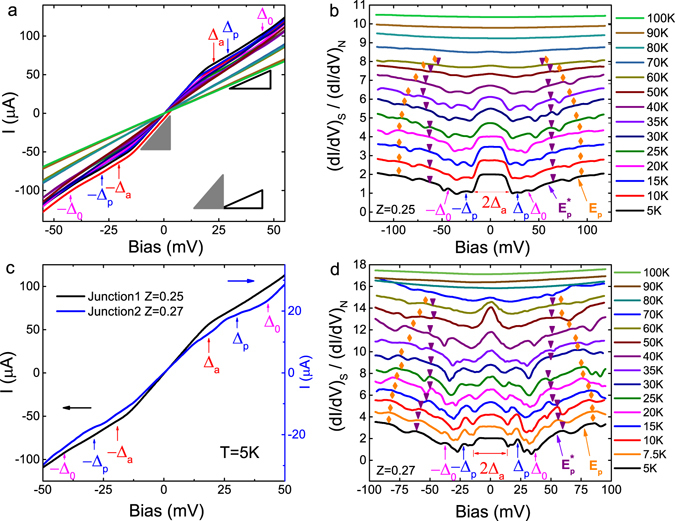
Highly transparent Bi-2212/1T-TaS_2_ junctions. (**a**) DC *I*–*V* curves of Junction-1, with BTK parameter Z = 0.25, at various temperatures from 5 K to 100 K. Δ_*a*_ (Red), Δ_*p*_ (Blue) and Δ_0_ (Magenta) respectively represent the induced superconducting gap on 1T-TaS_2_ side at interface, the depressed superconducting gap on Bi-2212 side at interface, and the intrinsic superconducting gap of Bi-2212. The hollow solid triangle indicates the normal current of the junction, where the normal conductance at low temperatures in the superconducting state is approximately equal to that of the normal state. The solid gray triangle indicates the excess current due to Andreev reflection at N-S interface, where the conductance within the Andreev reflection region amounts to nearly twice the normal state conductance well below *T*_*c*_. (**b**) Normalized AC differential conductance ${(dI/dV)}_{S}/{(dI/dV)}_{N}$ for Junction-1, where the differential conductance at 100 K is considered as the normal state conductance. The curves are shifted for clarity. (**c**) DC *I*–*V* curves of Junction-1 (Z = 0.25, black) and Junction-2 (Z = 0.27, blue) at 5 K. The induced superconducting gap Δ_*a*_ and excess current within ±Δ_*a*_ of Junction-2 are slightly lower relative to Junction-1. The normal conductance of Junction-2, around 0.62 *μ*A/mV, is lower than the normal conductance around 1.85 *μ*A/mV for Junction-1. (**d**) AC differential conductance ${(dI/dV)}_{S}/{(dI/dV)}_{N}$ for Junction-2, normalized by the differential conductance at 100 K. Curves are shifted for clarity. Note: for panels (**b**,**d**), the orange/purple markers and arrows are discussed in the text.

DC current-voltage *I*–*V* curves of Junction-1, with BTK parameter Z = 0.25, show clear zero-bias excess current relative to the normal current (indicated by the red vertical arrows labeling the induced gap, ±Δ_*a*_) as shown in Fig. 2(a) for temperatures from 5 K to 100 K. The slope within the excess current range from −20 meV to 20 meV at 5 K, as indicated by the solid gray triangle, is nearly twice the slope within the normal current range indicated by the solid hollow triangle. At temperatures well below the *T*_*c*_ (~85 K) of Bi-2212, the excess current in the vicinity of zero bias is approximately twice the normal current, and therefore attributable to Andreev reflection at the interface^29^, where the electrons injected from N side are reflected as holes with time reversal symmetry tracing the injected electrons’ track back into N side. To conserve current across the interface, Cooper pairs flow at the Fermi energy within S. These Cooper pairs have electron-like (ELQ) and hole-like (HLQ) quasiparticle character above/below the edges of the energy gap and the conversion process only occurs when the incident electrons on the N side lie within the conductance plateau defined over the energy range ±Δ_*a*_ shown in the normalized differential conductance plot of Fig. 2(b).

The two additional nonlinear features marked as blue and magenta vertical arrows in Fig. 2 are attributed respectively to the two superconducting gaps Δ_*p*_ and Δ_0_ mentioned at the beginning of this section. The AC differential conductance spectroscopies (*dI*/*dV*)_*S*_ of Junction-1, normalized by the normal state conductance (*dI*/*dV*)_*N*_ at 100 K, at various temperatures from 5 K to 100 K shown in Fig. 2(b) confirm the features observed from *I*–*V* curves. The proximity effect induced superconducting gap Δ_*a*_ = ±20 meV at 5 K on the 1T-TaS_2_ side of the interface delineates the voltage region within which Andreev reflection occurs. Additional gap features, relevant to the nonlinear features observed from *I*–*V* curves, are believed to be the density of states (DOS) features corresponding to the depressed superconducting gap Δ_*p*_ on the Bi-2212 side at the interface and the intrinsic superconducting gap Δ_0_ of Bi-2212. For Junction-1, the sizes of the two gaps are Δ_*p*_ = 28 meV and Δ_0_ = 40 meV respectively at 5 K. As temperature increases, the zero-bias conductance peak evolves from a flat mesa to a rounded hump while the peak’s width and height are depressed up to temperatures near 80 K. The gaps, Δ_*p*_ and Δ_0_ merge with increasing temperature until all gaps disappear near 80 K. Similar features are also observed on Junction-2 with BTK parameter Z = 0.27, as shown in Fig. 2(c) inset for DC *I*–*V* curves and (d) for AC differential conductance spectroscopies.

Both Junction-1 and Junction-2 show characteristics of highly transparent junctions, exhibiting high normal state conductance of 1.85 *μ*A/mV and 0.62 *μ*A/mV at 5 K respectively as shown in Fig. 2. As revealed by the conductance spectroscopies of Junction-2 (seen in Fig. 2(d)), the sizes of the three gaps Δ_0_ = 39 mV, Δ_*p*_ = 23 mV and Δ_*a*_ = 16 mV at 5 K are close to the values of Junction-1. The consistency of the Bi-2212 intrinsic superconducting gap size measured for Junction-1 and Junction-2 with previous *c*-axis point-contact tunneling studies on single crystal Bi-2212^26, 27^ is satisfying. Any variation of the differential conductance spectroscopies’ line-shape for Junction-1 and Junction-2 might be ascribed to the discrepancy of proximity regions’ width and scattering rate of quasiparticles at the vicinity of the interface^1, 3, 30–33^.

Andreev reflection such as observed here for our low-Z junctions (Junction-1 and Junction-2) exists at the normal metal-superconductor (N-S) interface, thereby indicating that the observed Andreev reflection feature on our Bi-2212/1T-TaS_2_ junctions implies at minimum a metallic component on the N side. We conclude that the metastable metallic phase of 1T-TaS_2_ residing in the Mott-CCDW state at low temperatures and revealed by STM studies^17–21^ is the requisite metallic state for proximity coupling to pristine 1T-TaS_2_, where the metallic phase has a smaller parameter *U*/*W* than the Mott-CCDW phase. Importantly, the Andreev enhanced zero-bias peak disappearing at high temperature around 80 K in Junction-1 and Junction-2 reflects *proximity induced high*-*T*_*c*_ *superconductivity* in the metastable metallic phase residing in the layers of 1T-TaS_2_ which are adjacent to the Bi-2212.

Besides the evidence for a robust superconducting proximity effect existing in our two low-Z Bi-2212/1T-TaS_2_ junctions, we find two dip-hump structures positioned at U_e–ph_ and ${{\rm{U}}}_{{\rm{e}}-{\rm{ph}}}^{\ast }$ for both junctions, which are indicated at their respective peaks (humps) by orange diamond and purple inverted triangles in Fig. 2(b,d). As suggested by neutron resonance and ARPES results^34, 35^, a single dip-hump structure in Bi-2212 probably stems from the electron-boson coupling, or combined electron-boson coupling and pseudogap^36^ corresponding to a boson mode energy Ω. As the *fingerprint* of a boson mode, the energy at the maximum slope of the dip-hump structure in conductance spectroscopies or the peak/dip in the *dI* ^2^/*dV* ^2^ spectra at positive/negative bias regions for NIS junctions is positioned at E_p_ = Δ_0_ + Ω^37–40^. Indicated by orange and purple arrows for the lowest temperatures in Fig. 2(b,c) respectively in the conductance spectroscopies, the determination of experimental values of E_p_ and ${{\rm{E}}}_{{\rm{p}}}^{\ast }$ from *dI* ^2^/*dV* ^2^ spectra are described in Fig. S.4 of Supplementary information. Consequently, with the information summarized in Table 1 at 5 K, the boson mode energies referred to the two dip-hump structures are Ω = E_p_ − Δ_0_ = 48 meV and ${{\rm{\Omega }}}^{\ast }={{\rm{E}}}_{{\rm{p}}}^{\ast }-{{\rm{\Delta }}}_{0}=24\,{\rm{meV}}$ for Junction-1 with ${{\rm{\Delta }}}_{0}=40\,{\rm{meV}}$; ${\rm{\Omega }}=40\,{\rm{meV}}$ and ${{\rm{\Omega }}}^{\ast }=17\,{\rm{meV}}$ for Junction-2 with ${{\rm{\Delta }}}_{0}=39\,{\rm{meV}}$. For intrinsic Bi-2212, the boson mode energy found in STM spectra is around 52 ± 8 meV^41^, leading us to conclude that the hump peaked at U_e–ph_ is from intrinsic Bi-2212 accompanied by a boson mode energy Ω, while the hump peaked at ${{\rm{U}}}_{{\rm{e}}-{\rm{ph}}}^{\ast }$ is a heretofore unseen hump due to another boson mode with lower energy Ω*. As temperature increases, the two humps merge to a single hump near 50–60 K for both junctions, a temperature somewhat more than the temperature near 40 K where Δ_0_ and Δ_*p*_ merge as shown in the differential conductance spectroscopies in Fig. 2(b,d).

**Table 1:** Parameters used in calculation (marked as “*theory*”) or from measurements (marked as “*exp*”) of the conductance spectroscopies at 5 K for various junctions.

	Z	${\tilde{{\boldsymbol{\sigma }}}}_{{\boldsymbol{N}}}$ *μ*A/mV	${{\boldsymbol{\Delta }}}_{{\bf{0}}}^{\exp }/{{\boldsymbol{\Delta }}}_{{\bf{0}}}^{{\boldsymbol{theory}}}$ meV	${{\boldsymbol{\Delta }}}_{{\boldsymbol{p}}}^{\exp }/{{\boldsymbol{\Delta }}}_{{\boldsymbol{p}}}^{{\boldsymbol{theory}}}$ meV	${{\boldsymbol{\Delta }}}_{{\boldsymbol{a}}}^{\exp }/{{\boldsymbol{\Delta }}}_{{\boldsymbol{a}}}^{{\boldsymbol{theory}}}$ meV	Γ_0_ meV	Γ_*p*_ meV	Γ_*a*_ meV	U_e–ph_/E_p_ meV	${{\bf{U}}}_{{\bf{e}}-{\bf{ph}}}^{{\boldsymbol{\ast }}}/{{\bf{E}}}_{{\bf{p}}}^{{\boldsymbol{\ast }}}$ meV	$\frac{{{\boldsymbol{d}}}_{{\boldsymbol{S}}}}{{{\boldsymbol{\xi }}}_{{\bf{0}}}}/\frac{{{\boldsymbol{d}}}_{{\boldsymbol{vdW}}}}{{{\boldsymbol{\xi }}}_{{\bf{0}}}}/\frac{{{\boldsymbol{d}}}_{{\boldsymbol{N}}}}{{{\boldsymbol{\xi }}}_{{\bf{0}}}}$
Junction-1	0.25	1.85	40/41.0	28/26.8	20/19.1	0.8	2.7	0.1	95/88	65/64	5.5/2/10
Junction-2	0.27	0.62	39/39.6	23/22.8	16/16.5	0.8	0.2	0.1	83/79	63/56	5/1.9/8
Junction-3	0.5	0.21	38/42.3	21/18.5	21/17.3	0.6	9.1	5.2	93/89	66/62	4.4/1.6/2
Junction-4	0.8	—	39/39.2	23/23.3	23/22.2	2.1	2.3	2.2	—	—	4/1.2/0.5
Junction-5	2.5	—	41/40.3	—	—	8.1	—	—	—	—	—
Bi-2212/graphite	1	55.62	42/40.5	—	—	6.1	—	—	—	—	—

Z: BTK parameter; ${\tilde{\sigma }}_{N}$: normal conductance from measurement; Δ_0_: Bi-2212 intrinsic superconducting gap; Δ_*p*_: suppressed superconducting gap on S side; Δ_*a*_: proximity induced superconducting gap on N side; Γ_0_: quasiparticle lifetime parameter on S side; Γ_*p*_: quasiparticle lifetime parameter at interface on S side; Γ_*a*_: quasiparticle lifetime parameter at interface on N side; ${{\rm{U}}}_{{\rm{e}}-{\rm{ph}}}/{{\rm{U}}}_{{\rm{e}}-{\rm{ph}}}^{\ast }$: position of higher/lower energy scaled hump on conductance spectroscopy. ${{\rm{E}}}_{{\rm{p}}}/{{\rm{E}}}_{{\rm{p}}}^{\ast }$: position of higher/lower energy scaled peak/dip in positive/negative voltage regions of *d* ^2^ *I*/*dV* ^2^. *d*_*S*_/*d*_*vdW*_/*d*_*N*_: thickness of proximity region on S side, Van der Waals stacking distance and thickness of proximity region on N side.

#### Two boson modes

**Figure 3:**
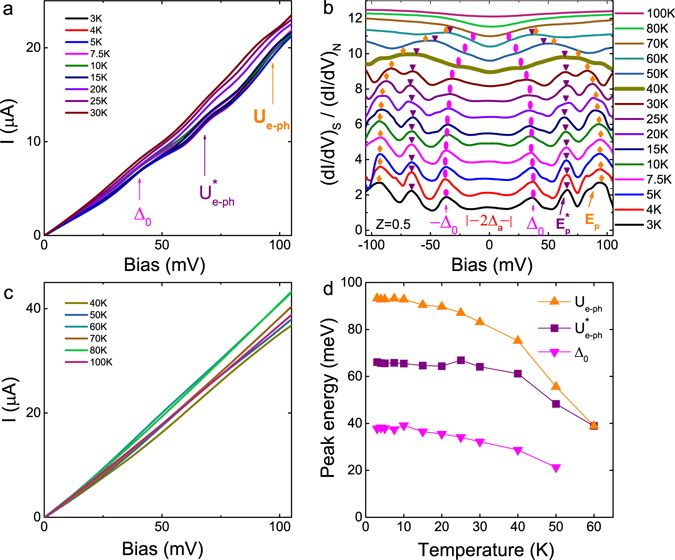
Bi-2212/1T-TaS_2_ junction with BTK Z = 0.5 (Junction-3). (**a**) DC *I*–*V* curves of Junction-3, with BTK parameter Z = 0.5, at various temperatures from 3 K to 30 K, well below the *T*_*c*_ of Bi-2212. The Bi-2212 intrinsic superconducting gap Δ_0_ is indicated by a magenta vertical arrow. U_e–ph_ and ${{\rm{U}}}_{{\rm{e}}-{\rm{ph}}}^{\ast }$ correspond to the peak energy positions of two nonlinear features due to the two boson modes measured with respect to Δ_0_. The normal conductance decreases with temperature, where the normal conductance at 5 K for Junction-3 is around 0.21 *μ*A/mV. (**b**) Normalized AC differential conductance ${(dI/dV)}_{S}/{(dI/dV)}_{N}$ for Junction-3. The differential conductance at 100 K manually scaled into the range of the conductance at one specific temperature serves as the normal conductance. Relative to the NS Andreev reflection feature seen in Junction 1 and 2, the zero-bias excess differential conductance peak is significantly reduced and almost disappears at around 40 K. The Bi-2212 intrinsic superconducting gap Δ_0_ and the humps referring to boson modes with energies Ω* and Ω are indicated respectively by solid magenta ellipses, inverted purple triangles and orange diamonds. Positions of E_p_ and ${{\rm{E}}}_{{\rm{p}}}^{\ast }$ are marked by orange and purple arrows. The curves are shifted for clarity. (**c**) DC *I*–*V* curves of Junction-3 at various temperatures from 40 K to 100 K. The conductance in the normal state at 100 K is around 0.4 *μ*A/mV. The temperature dependence of the conductance for Junction-3 evolves consistently with intrinsic 1T-TaS_2_ at temperatures from 3 K to 100 K shown in Fig. S.1(b). (**d**) Temperature dependence of the two humps’ peak positions and the Bi-2212 intrinsic superconducting gap.

Our Bi-2212/1T-TaS_2_ Junction-3, with slightly lower transparency (BTK parameter Z = 0.5), offers more information about the dip-hump structures observed in Junction-1 and Junction-2. As shown in Fig. 3(a), the DC *I*–*V* curves at various temperatures (3 K to 30 K) well below Bi-2212 *T*_*c*_ show several nonlinear features. Besides the nonlinear feature of the Bi-2212 intrinsic superconducting gap Δ_0_ indicated by the magenta vertical arrow, the other two nonlinear features taking place above Δ_0_, as indicated by the purple and orange vertical arrows, are also clearly observed. Junction-3 with lower normal conductance around 0.21 *μ*A/mV at 5 K than Junction-1 and Junction-2, however, demonstrates the normal conductance varies with temperatures indicating the 1T-TaS_2_ for Junction-3 might behave more like a Mott-CCDW material rather than the metastable metallic material in the more transparent junctions. The temperature dependence of normal conductance in Junction-3, referring to a less metallic NS junction, probably originates from the carrier delocalization in the Mott-insulating state in 1T-TaS_2_ with increasing temperature^13–16^.

The normalized AC differential conductance spectroscopies ${(dI/dV)}_{S}/{(dI/dV)}_{N}$ of Junction-3, obtained using the method described in the Methods section, at various temperatures from 3 K to 100 K are shown in Fig. 3(b). A strongly suppressed amplitude of the zero-bias peak for Junction-3 relative to Junction-1 and Junction-2 is observed. Such strong suppression suggests a stronger scattering rate or shorter quasiparticle lifetimes in the proximity-effect-induced superconducting region on the 1T-TaS_2_ side at interface. The width of the zero-bias peak decreases with increasing temperature, and is totally suppressed near 40 K. Meanwhile, the feature representing the depressed superconducting gap Δ_*p*_ is clearly not observed, as seen in Fig. 3(b), which we ascribe to the closer proximity between Δ_*p*_ and Δ_*a*_ and temperature broadening. For Junction-3, the measured superconducting gap Δ_0_ around 38 meV at 5 K is slightly smaller but still consistent with the highly transparent Junction-1 and Junction-2. Notably, the width of zero-bias peak is around 21 meV, which is not strongly depressed relative to Junction-1 and Junction-2.

Sharper and clearer signatures of two dip-hump structures are observed in Junction-3. The conductance spectroscopy indicates the hump (peak marked by the orange diamond) due to the boson mode energy Ω has a broader width than the hump (peak marked by the purple inverted triangle) due to the boson mode energy Ω*. The two humps of Junction-3 at 5 K which are peaked at the energy ${{\rm{U}}}_{{\rm{e}}-{\rm{ph}}}=93\,{\rm{meV}}$ and ${{\rm{U}}}_{{\rm{e}}-{\rm{ph}}}^{\ast }=66\,{\rm{meV}}$, as well as the maximum slope positions of dip-hump structures E_p_ = 89 meV and ${{\rm{E}}}_{{\rm{p}}}^{\ast }=62\,{\rm{meV}}$ are listed in Table 1, which correspond to the boson mode energies ${\rm{\Omega }}={{\rm{E}}}_{{\rm{p}}}-{{\rm{\Delta }}}_{0}=89-38=51\,{\rm{meV}}$ and ${{\rm{\Omega }}}^{\ast }={{\rm{E}}}_{{\rm{p}}}^{\ast }-{{\rm{\Delta }}}_{0}=62-38=24\,{\rm{meV}}$. More details about the temperature evolution (at temperatures below Bi-2212 *T*_*c*_) of the Bi-2212 superconducting gap Δ_0_ and two humps are presented in Fig. 3. Similar to the highly transparent Junction 1 and 2, the two humps merge toward each other with increasing temperature, and almost merge into a single hump at an energy position of ${{\rm{U}}}_{{\rm{e}}-{\rm{ph}}}={{\rm{U}}}_{{\rm{e}}-{\rm{ph}}}^{\ast }=39\,{\rm{meV}}$ at around 60 K. The zero-bias conductance peak is totally suppressed at a lower temperature around 40 K instead of 80 K as in Junction 1 and 2; however, still indicating proximity-induced high-*T*_*c*_ superconductivity in 1T-TaS_2_.

### Theoretical modeling and results

**Figure 4:**
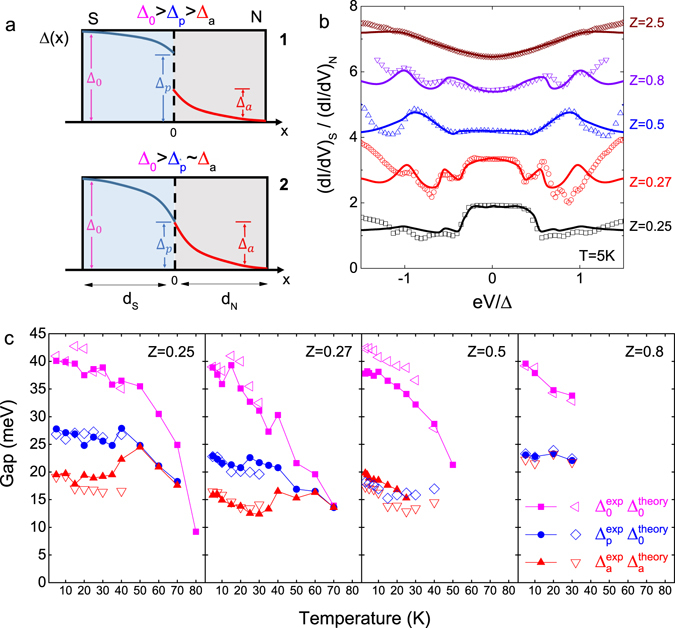
Superconducting proximity effect and theoretical modeling. (**a**) Schemes of NS junction with superconducting proximity effect. Δ_0_, Δ_*p*_ and Δ_*a*_ correspond to the intrinsic superconducting gap, depressed superconducting gap on S side at interface and induced superconducting gap on N side at the interface due to the superconducting proximity effect. Upper scheme: ${{\rm{\Delta }}}_{0} > {{\rm{\Delta }}}_{p} > {{\rm{\Delta }}}_{a}$; Lower scheme: ${{\rm{\Delta }}}_{0} > {{\rm{\Delta }}}_{p}\sim {{\rm{\Delta }}}_{a}$. (**b**) Calculated (colored solid lines) and measured (hollow symbols) normalized differential conductance ${(dI/dV)}_{S}/{(dI/dV)}_{N}$ at 5 K for various Bi-2212/1T-TaS_2_ junctions, with BTK parameters Z = 0.25, 0.27, 0.5, 0.8 and 2.5. The junction with Z = 2.5 does not show any superconducting proximity effect at the NS interface. Curves are shifted for clarity. (**c**) Temperature dependence of Δ_0_, Δ_*p*_ and Δ_*a*_ for various Bi-2212/1T-TaS_2_ junctions: Junction-1, Z = 0.25; Junction-2, Z = 0.27; Junction-3, Z = 0.5; Junction-4, Z = 0.8. More details are discussed in the text. The gaps used in the theoretical calculation of ${(dI/dV)}_{S}/{(dI/dV)}_{N}$ are marked as large-sized hollow symbols, and the gaps from measurements are marked as small-sized solid symbols.

The superconducting proximity effect distinguishes two major regimes at the interface of NS or NIS junctions^1, 3^. The NS interface for electrons with energy *E* < Δ_*a*_ injected from the N side is the boundary between the induced superconducting region and the normal material on the N side, whereas electrons within energy Δ_*a*_ < *E* < Δ_*p*_ and Δ_*p*_ < *E* < Δ_0_ will experience the NS interface from the S side. Two schemes of a proximity junction with thicknesses of the proximity regions on the S and N sides respectively marked as *d*_*S*_ and *d*_*N*_ are depicted in Fig. 4(a). Schemes Nos. 1 and 2 correspond respectively to the cases Δ_0_ > Δ_*p*_ > Δ_*a*_ and Δ_0_ > Δ_*p*_ ~ Δ_*a*_.

The *c*-axis conductance characteristics of various junctions, including Bi-2212/1T-TaS_2_ and Bi-2212/graphite junctions, are calculated based on an extended BTK model of the tunneling spectrum for anisotropic superconductors^22^. For Bi-2212, with *d*-wave symmetry of the superconducting gap^42–44^, the electron-like quasiparticle (ELQ“+”) and hole-like quasiparticle (HLQ“–”) experience the same pairing potential, $|{{\rm{\Delta }}}_{+}|=|{{\rm{\Delta }}}_{-}|={{\rm{\Delta }}}_{0}\,\cos \,\mathrm{(2}\alpha )$, where the angle *α* in the plane perpendicular to the *c*-axis is a measure of the gap lobe’s orientation and global phase ${\varphi }_{+}={\varphi }_{-}=0$ along the *c*-axis. Then, the *c*-axis normalized differential conductance ${(dI/dV)}_{S}/{(dI/dV)}_{N}$ is expressed as

1 $$\frac{{(dI/dV)}_{S}}{{(dI/dV)}_{N}}(V)={\int }_{-\infty }^{+\infty }\frac{\partial {f}_{0}(E-eV)}{\partial (eV)}{\sigma }_{T}(E)dE,$$

where *f*_0_(*E*) is the Fermi-Dirac distribution at temperature *T*. The dimensionless tunneling conductance at an energy *E* away from *E*_*F*_ is described as

2 $${\sigma }_{T}(E)=\frac{{\int }_{{\rm{\Omega }}}\,[1+{R}_{eh}^{2}(E)-{R}_{ee}^{2}(E)]\,{\sigma }_{N}\,\cos \,\theta d{\rm{\Omega }}}{{\int }_{{\rm{\Omega }}}\,{\sigma }_{N}\,\cos \,\theta d{\rm{\Omega }}}.$$

Equation (2) corresponds to a semi-spherical solid angle integration over the Fermi surface of *d*-wave superconductors. In the case of no mismatch of the Fermi level across the interface, the dimensionless normal conductance or transparency of the interface is ${\sigma }_{N}=\frac{1}{1+{Z}^{2}}$ as mentioned earlier. The rate of Andreev reflection (AR) *R*_*eh*_(*E*) and ordinary reflection (OR) *R*_*ee*_(*E*), based on the extended BTK model^22^, are expressed in equation (S.5) in Supplementary information. Additionally, by simply adding an imaginary energy term −*i*Γ (as the quasiparticle lifetime parameter) to *E* → *E* − *i*Γ and ${{\rm{\Omega }}}_{\pm }\to \sqrt{{(E-i{\rm{\Gamma }})}^{2}-{{\rm{\Delta }}}_{0}^{2}}$, the smearing effect on tunneling spectroscopy due to quasiparticle life time *τ*_*R*_ or scattering rate 1/*τ*_*R*_ is quantitatively described^33, 45^.

The calculated conductance spectroscopies for various junctions show good agreement with measurement results, as shown in Fig. 4(b) for various junctions at 5 K, as well as the temperature-dependent conductance spectroscopies below Bi-2212 *T*_*c*_ for Bi-2212/1T-TaS_2_ and Bi-2212/graphite junctions seen in Fig. S.3(a–e). Figure 4(c) also compares the temperature dependent gaps (Δ_0_, Δ_*p*_ and Δ_*a*_) with theoretical calculations for various Bi-2212/1T-TaS_2_ junctions. These calculations were only carried out up to 40 K because of high temperature smearing effects. The parameters used in calculation of the conductance characteristics at 5 K for various junctions are listed in Table 1. More details on theoretical modeling are described in Supplementary information (Section: Supplementary Discussion–Theoretical Modeling).

## Discussion

**Figure 5:**
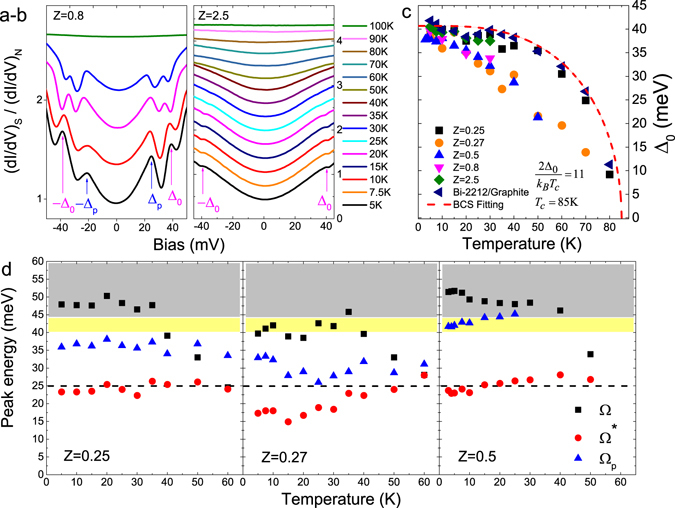
Junctions with lower transparencies, measured Bi-2212 intrinsic superconducting gap and boson mode energies in junctions. (**a**,**b**) Normalized differential conductance spectroscopies ${(dI/dV)}_{S}/{(dI/dV)}_{N}$ at various temperatures (5 K to 100 K) for Junction-4 (left, BTK Z = 0.8) and Junction-5 (right, BTK Z = 2.5). Curves are shifted for clarity. (**c**) Temperature dependent Bi-2212 intrinsic superconducting gap Δ_0_ measured from various junctions, including Bi-2212/1T-TaS_2_ and Bi-2212/graphite junctions. The red dashed line indicates the BCS fitting with a gap ratio $2{{\rm{\Delta }}}_{sc}/{k}_{B}{T}_{c}=11$. (**d**) Temperature dependence of boson mode energy ${\rm{\Omega }}={{\rm{E}}}_{{\rm{p}}}-{{\rm{\Delta }}}_{0}$, ${{\rm{\Omega }}}^{\ast }={{\rm{E}}}_{{\rm{p}}}^{\ast }-{{\rm{\Delta }}}_{0}$ and ${{\rm{\Omega }}}_{p}={{\rm{E}}}_{{\rm{p}}}^{\ast }-{{\rm{\Delta }}}_{p}$ for Junction-1–3. At 5 K, Ω, Ω* and Ω_*p*_ are: 48 meV, 24 meV and 36 meV for Junction-1; 40 meV, 17 meV and 33 meV for Junction-2; 51 meV, 24 meV and 41 meV for Junction-3. Gray background indicates the energy range from 44 meV to 60 meV, yellow background indicates the energy range from 40 meV to 44 meV, and black dashed line indicates the energy level of 25 meV. Note, the Ω_*p*_ for Junction-3 is calculated by the predicted value based on the modeling due to the difficulty of distinguishing Δ_*p*_ and Δ_*a*_ in Junction-3.

The normalized differential conductance plots, as shown in Fig. 5(a), at temperatures from 5 K to 30 K for Bi-2212/1T-TaS_2_ Junction-4 with BTK parameter Z = 0.8 exhibit the proximity effect described by Scheme No. 2 in Fig. 4(a). For this case there is no discontinuity in the gap at the interface (i.e., Δ_*p*_ = Δ_*a*_). For even lower transparency interfaces, such as Junction-5 with BTK parameter Z = 2.5 shown in Fig. 5(b), any remnants of a superconducting proximity effect with markers at Δ_*p*_ and Δ_*a*_ have disappeared. To briefly summarize, for relatively high transparent Bi-2212/1T-TaS_2_ Junction 1–4, the superconducting proximity effect is clearly experimentally observed (seen in Figs 2, 3 and 5(a)) via *c*-axis conductance spectroscopies. The temperature dependence of the gaps Δ_0_, Δ_*p*_ and Δ_*a*_ for Junction 1–4, displayed in the four panels of Fig. 4(c), show a merging of Δ_*p*_ and Δ_*a*_ with decreasing transparency (or increasing Z) of the interface and increasing temperature.

**Figure 6:**
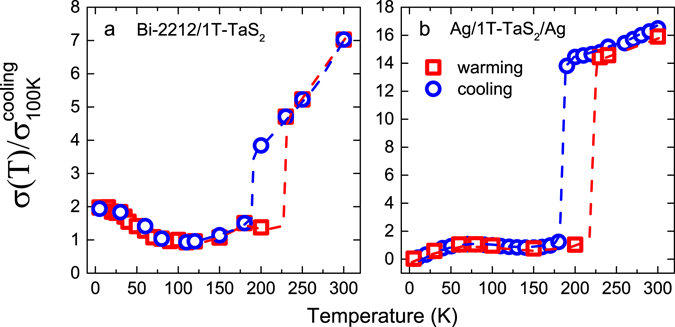
Normalized zero-bias conductance for (**a**) Bi-2212/1T-TaS_2_ Junction-1 (Z = 0.25) and (**b**) Ag/1T-TaS_2_/Ag junction plotted as a function of temperature under a cooling/warming cycle. The reference conductance is the zero-bias conductance at 100 K on the cooling cycle. The red and blue dashed lines indicate the CCDW-NCCDW transition of 1T-TaS_2_.

From the linear DC *I*–*V* characteristics (seen in Figs 2(a) and S.5(a,b)), the normalized zero-bias conductance (NZBC) for Junction-1 with Z = 0.25 divided by the conductance at 100 K is calculated and shown in Fig. 6(a) as a function of temperature for the cooling cycle. At high temperatures above *T*_*c*_ where the Bi-2212 is in the normal state, hysteresis dominates in the temperature range of 180 K to 230 K as it does in the four-terminal temperature-dependent resistance of a pristine 1T-TaS_2_ flake shown in Supplementary Fig. S.1(b).

For additional insight, we use a back-to-back structured Ag/1T-TaS_2_/Ag trilayer junction to detect the perpendicular transport characteristic of a metal/1T-TaS_2_ junction. The linear *I*–*V* characteristics are shown in Fig. S.5(c,d). As shown in Fig. 6(b), the temperature-dependent NZBC of Ag/1T-TaS_2_/Ag reveals the signature of the CDW transition in 1T-TaS_2_ by a strong suppression of the NZBC when 1T-TaS_2_ transits from a metallic NCCDW phase to a Mott-CCDW phase. In this case the metal/1T-TaS_2_ junction is expected to include a metal/semiconductor barrier owing to the opened Mott gap of 1T-TaS_2_ at low temperatures. Thus, the observed hysteresis in Junction-1 suggests the existence of CDW transitions when Bi-2212 is intimately contacted to 1T-TaS_2_ either as a superconductor or a normal metal. In both cases the 1T-TaS_2_ has clearly converted to a Mott-CCDW phase at low temperatures. Moreover, the NZBC of Junction-1 in panel (a) has increased with decreasing temperature up to almost 2 times that of the referenced 100 K conductance due to the Andreev reflection contributions at base temperature 5 K. Accordingly, the superconducting proximity effect and the CDW transition are simultaneously present in the same sample. We also note an obviously suppressed amplitude of the hysteresis window for Junction-1 compared to the Ag/1T-TaS_2_/Ag junction. Such a signature of suppression reveals the more metallic nature of 1T-TaS_2_ when in contact with Bi-2212.

Consequently, the superconducting proximity effect observed in our highly transparent Bi-2212/1T-TaS_2_ junctions strongly suggests that high-*T*_*c*_ superconductivity forms within the metastable metallic phase with a smaller parameter *U*/*W*, residing in the Mott-CCDW phases located in the topmost layers of 1T-TaS_2_. The different heights and widths of the zero bias peak on the conductance characteristics for various Bi-2212/1T-TaS_2_ junctions with different transparencies might be ascribed to the Andreev reflection corresponding to different configurations of Mott-CCDW phase and metallic phase in 1T-TaS_2_ (See Supplementary information for more discussion). The curved and depressed zero-bias conductance peak (seen in Figs 2(b,d) and 3(b)) corresponds to the smearing effect with increasing quasiparticle scattering rate^33, 45^, Accordingly, it is no surprise that the quasiparticle lifetime parameters Γ_*a*_ at the interface on the N side (see Table 1) are lower by more than a factor of ten for the high transparency Junction 1 and 2 than they are for the lower transparency Junction 3 and 4.

We rule out alternative interpretations with the following arguments: Firstly, the Bi-2212 intrinsic superconducting gap Δ_0_ measured from various Bi-2212/1T-TaS_2_ and Bi-2212/graphite junctions shows consistency of the results at temperatures well below Bi-2212 *T*_*c*_, with the BCS gap ratio 2Δ_*sc*_/*k*_*B*_ *T*_*c*_ to be around 10.4–11.5 (seen in Fig. 5(c)) in good agreement with previous works on intrinsic Bi-2212^26, 27^. Moreover, less dependence of the proximity induced gap Δ_*a*_ on temperature (seen in Fig. 4(c)) rules out a contribution to the differential conductance from *Andreev bound states caused by planar geometry or surface roughness* ^46–48^. (Here, we claim the larger deviation of measured superconducting gaps among various junctions at higher temperatures close to the *T*_*c*_ of Bi-2212 is probably due to distortion of gap features on conductance spectroscopies by stronger scattering and temperature smearing effects, which could be self-consistently verified by the smaller deviations of gap parameters used in theoretical results shown in Fig. S3(f)). Secondly, though there is a strong suppression of amplitude for the zero bias conductance peak for various junctions (almost two times the normal conductance in Junction-1 and Junction-2, whereas 30% larger than normal conductance in Junction-3), less temperature dependence of the wide induced gap Δ_*a*_ (41% to 55%Δ_0_ on 1T-TaS_2_ side as shown in Fig. 4(c)) rules out the feature of the *complex Andreev reflection due to the phase conjugation of electrons and holes* predicted for superconductor–semiconductor interfaces^49^. Thirdly, there is no dominant feature of periodic conductance peaks observed in any of our Bi-2212/1T-TaS_2_ junctions, which rules out the possibility that our observed proximity feature is related to *McMillan–Rowell oscillation* observed in some normal metal/cuprate junctions^31, 50–53^. Lastly, with a very short *c*-axis coherence length (~1 Å^54^) in intrinsic Bi-2212, the absence of evidence for Josephson junctions intrinsically formed by superconducting CuO_2_ and non-superconducting Bi–O and Sr–O layers^55, 56^ observed at temperatures well below *T*_*c*_ rules out the explanation of the *superconducting proximity effect masquerading as interlayer tunneling* within the Bi-2212.

In Section IIB-2 we discussed the presence of two dip-hump structures, U_e–ph_ and ${{\rm{U}}}_{{\rm{e}}-{\rm{ph}}}^{\ast }$ which when associated with peak/dip features in the *dI* ^2^/*dV* ^2^ spectra at positive/negative bias regions defined respectively the energies E_p_ and ${{\rm{E}}}_{{\rm{p}}}^{\ast }$. We then referenced these features to the intrinsic Bi-2212 gap edge Δ_0_ using the relations ${\rm{\Omega }}={{\rm{E}}}_{{\rm{p}}}-{{\rm{\Delta }}}_{0}$ and ${{\rm{\Omega }}}^{\ast }={{\rm{E}}}_{{\rm{p}}}^{\ast }-{{\rm{\Delta }}}_{0}$ and find that the temperature-dependent boson mode energy Ω is consistent with the STM spectrum on intrinsic Bi-2212^41^. This picture is incomplete however without comparing the energy of the second feature referenced to the intrinsic gap, ${{\rm{\Omega }}}^{\ast }={{\rm{E}}}_{{\rm{p}}}^{\ast }-{{\rm{\Delta }}}_{0}$ and alternatively referenced to the reduced gap at the interface, Δ_*p*_, using the relation ${{\rm{\Omega }}}_{p}={{\rm{E}}}_{{\rm{p}}}^{\ast }-{{\rm{\Delta }}}_{p}$, revealing a slightly larger energy scale than Ω* both of which are derived from ${{\rm{E}}}_{{\rm{p}}}^{\ast }$. For Junction 1–3 these three boson modes, Ω, Ω* and Ω_*p*_ are plotted in Fig. 5(d) as a function of temperature.

Here, we provisionally assume the two dip–hump structures in conductance spectroscopies, observed in Junction-1–3, are due to self energy effects related to electron-boson interaction where phonons serve as the relevant bosons. We then infer that the “glue” assisting the strong-coupled pairing of electrons responsible for the high-*T*_*c*_ superconductivity in 1T-TaS_2_ arises from phonons. The key point, here, is whether the additional hump peaked at ${{\rm{U}}}_{{\rm{e}}-{\rm{ph}}}^{\ast }$ is the hump corresponding to the suppressed superconducting gap Δ_*p*_ because of a boson mode energy Ω_*p*_ representing the difference between the junction interface and bulk Bi-2212, or a hump related to intrinsic superconducting gap Δ_0_ due to a boson mode energy Ω* incorporating the effect of interplay between electron-electron and electron-phonon interactions in 1T-TaS_2_ on the density of states. For all junctions, the boson mode energy Ω at low temperatures is within the range of 40 meV to 60 meV (as indicated by the gray background) as well as being less temperature dependent. The results are consistent with the STM spectrum on intrinsic Bi-2212^41^. The yellow background demarcates the possible reduction (~4 meV^41^) due to the substitution of ^16^O by ^18^O in Bi-2212 crystals. Slightly lower energy Ω_*p*_ relative to Ω is observed in all Junction 1–3. On the other hand, all junctions reveal a nearly temperature-independent boson mode energy Ω* around 25 meV indicated by the horizontal black dashed line (except Junction-2 over limited temperatures). The magnitude of Ω* is in good agreement with the energy scale of an observed 25 meV infrared optical phonon^57^ corresponding to a CDW near the same energy^58^ that provides insight into the disorder–induced quasimetallic phase of 1T-TaS_2_ residing in the Mott-CCDW phase. Additionally, the observed evolution of broader (Junction-1–2 as seen in Fig. 2) to sharper (Junction-3 as seen in Fig. 3) line-shape of the hump peaked at ${{\rm{U}}}_{{\rm{e}}-{\rm{ph}}}^{\ast }$ is consistent with current evidence and explanations of the transition from a Mott-insulating to a metallic phase in 1T-TaS_2_ due to reduced Coulomb interaction *U* and broadened band width *W* of lower Hubbard band^17–21, 58^.

In conclusion, we have used differential conductance spectroscopy of Bi-2212/1T-TaS_2_ junctions with varying transparencies to find high-*T*_*c*_ superconductivity induced within pristine 1T-TaS_2_ by the proximity effect. The CDW order in the 1T-TaS_2_ appears to play an important role firstly by coexisting with an unexpected and surprisingly high *T*_*c*_ of the proximity gap in the 1T-TaS_2_ and secondly by revealing a heretofore unseen ancillary dip-hump feature that accompanies a primary dip-hump feature corresponding to a boson (phonon) mode Ω previously seen in STM work on intrinsic Bi-2212. This second dip-hump feature is clearly related to the proximity of the CDW dominated 1T-TaS_2_ and implies one of two possible boson modes depending on whether the dip-hump feature is referenced to the reduced gap Δ_*p*_ or the intrinsic gap Δ_0_ at the interface. In the former case the temperature-dependent boson mode ${{\rm{\Omega }}}_{p}={{\rm{E}}}_{{\rm{p}}}^{\ast }-{{\rm{\Delta }}}_{p}$ has values in the range 30–45 meV, somewhat less than the 40–50 meV range shown in Fig. 5(c) for Ω. The energy Ω_*p*_ can probably be interpreted as the mode relating to the gap Δ_*p*_ in the same manner as the mode Ω is related to the intrinsic gap Δ_0_. In the latter case however the temperature dependent boson mode ${{\rm{\Omega }}}^{\ast }={{\rm{E}}}_{{\rm{p}}}^{\ast }-{{\rm{\Delta }}}_{0}$ has values in the range 20–25 meV which is close to the infrared active phonon mode associated with the CDW in 1T-TaS_2_ as measured by infrared reflectance^57^. This more plausible latter interpretation provides independent evidence that the phonon associated with the CCDW phase coexists with and may even enhance the superconductivity in pristine 1T-TaS_2_. However, both of the above scenarios describing the second dip–hump structure provide reasonable rationales for the occurrence of a high-*T*_*c*_ proximity effect in Bi-2212/1T-TaS_2_ junctions. Our work posits a mutual interaction of the CCDW and superconducting order parameters in the interfacial region of Bi-2212/1T-TaS_2_ contacts, thereby revealing rich phenomenology and confirming a strong interplay between high *T*_*c*_ superconductivity and CDW order which is only beginning to be understood.

## Methods

### Sample fabrication

High quality, optimally doped single crystals of Bi_2_Sr_2_CaCu_2_O_8+*δ*_ (Bi-2212) were synthesized using the method of Mitzi *et al*.^59^, with the modification that a Pt crucible was used in place of an alumina one. This avoided possible contamination of the melt via reaction with the crucible walls. Single crystals of 1T-TaS_2_ flakes were prepared using iodine vapor transport^60^. The transport measurements shown in Fig. S1 verify the critical superconducting temperature of Bi-2212 to be 85 K, and the CCDW-NCCDW transition temperatures for cooling and warming processes of 1T-TaS_2_ to be 180 K and 230 K respectively. Prior to junction fabrication, thick flake Bi-2212 (thickness around 10 *μm* was mechanically exfoliated via Nitto-REVALPHA thermal release tape. Then, thick flake Bi-2212 was transferred to a clean glass substrate by thin double sided tape. Thin flake Bi-2212 (0.5 to 2 *μm*) was then cleaved via Scotch tape. A cleaved thin flake of 1T-TaS_2_ (2 to 5 *μm*) was immediately placed against the cleaved surface of Bi-2212 after similar mechanical exfoliation. The two cleaved flakes strongly adhere to each other via Van der Waals forces, naturally forming high quality NIS or NS heterostructures. Junctions with different transparencies, characterized with theoretical modeling by the extended BTK model described in the manuscript, were randomly achieved. No significant dependence of the transparency upon area of junction or thickness of flake was recognized. All steps of fabrication were performed in a dry atmosphere, and all cleaved surfaces of thin flake Bi-2212 and 1T-TaS_2_ were clean and flat. Based on multiple measured local-areas on the exfoliated pieces, the mean roughness for both materials is within 2 Å as indicated by the AFM images shown in Fig. 1(c)). Also, to implement four-terminal *c*-axis differential conductance measurements, the thin flake Bi-2212 and 1T-TaS_2_ were fashioned into narrow rectangular shapes oriented perpendicular to each other with an overlap area around 0.2 mm^2^, as shown in Fig. 1(d).

### Experimental measurements set-up

All measurements including the four-terminal AC differential conductance measurements and the DC current-voltage (*I*–*V*) measurements were performed over a wide range of temperature (2.5 K to 120 K) using a Quantum Design Physical Properties Measurement System (PPMS). Samples were mounted on a commercial PPMS puck and all measurements were performed in a low-noise screen room. For AC measurements, we used 23.3 Hz as the AC output frequency. The DC source voltage was supplied by a Keithley 2400 source meter and the AC source was supplied by a Agilent 33120 A AC generator with Δ*V* ~ 0.2 mV. The DC and AC source signals were added using a homemade DC + AC adder and then applied to the junction. The DC bias across the junctions was measured with HP 3456A multimeter, the AC voltage signal with a SR830 DSP lock-in amplifier, and the AC current signal with a second SR830 lock-in amplifier after converting the current to a voltage using a SR570 current preamplifier.

### Data normalization

For non-ideal NS junctions which have different density of states on the N and S sides, the conductance at one specific temperature cannot be simply normalized because of temperature dependence of the normal conductance. Thus, we normalized the AC differential conductance of non-ideal NS junctions, such as Junction-3, Junction-4 and Junction-5 discussed in the manuscript, by using the conductance at 100 K manually scaled into the range of the normal conductance at one specific temperature as the effective normal conductance. For instance, in Junction-3 the normal conductance at 5 K is around 0.2 *μ*A/mV and the conductance of the normal state at 100 K is around 0.4 *μ*A/mV. Hence, the ratio factor *r*_*eff*_ is the ratio of the normal conductance at 5 K to conductance at 100 K, which is around 0.5. Then, the normalized differential conductance spectroscopy at 5 K can be determined by multiplying a ratio factor *r*_*eff*_ when calculating the division of (*dI*/*dV*)_*S*_ by (*dI*/*dV*)_*N*_.

### Data availability

The datasets generated during and/or analysed during the current study are available from the corresponding author on reasonable request.

## Electronic supplementary material

Supplementary Material
