# Supplementary material for: Bi-2212/1T-TaS2 Van der Waals junctions: Interplay of proximity induced high-Tc superconductivity and CDW order
Source: Sci Rep. 2017 Jul 5;7:4639. doi: 10.1038/s41598-017-04645-1 (PMC5498642; doi:10.1038/s41598-017-04645-1)
Supplement: Supplementary file 1 — Supplementary Material [file 41598_2017_4645_MOESM1_ESM.pdf]

Bi-2212/1T-TaS_2 Van der Waals junctions: Interplay of proximity induced high-T_c superconductivity and CDW order —Supplementary Information

Ang J. Li, Xiaochen Zhu, G. R. Stewart and Arthur F. Hebard

Department of Physics, University of Florida, Gainesville, FL 32611, USA

(Dated: April 21, 2017)

CHARACTERIZATION OF INTRINSIC CRYSTALS

Experimental Method

Prior to transport measurement, single crystal Bi-2212 and 1T-TaS_2 samples (crystal structure shown in Fig.1(a) in manuscript) were first mechanically exfoliated in a dry atmosphere to produce thick flakes with thickness around 10 μm. Then, four-terminal transport measurements (contact configuration is shown in Fig S. 1(a) inset) were carried out using a SR830 DSP Lock-in Amplifier with an AC voltage of 0.5 V, frequency of 526 Hz and input impedance of 100 kΩ. The four-terminal configuration was constructed by gold wires and SPI silver paste. The temperature range for transport measurements is up to 300 K.

Bi-2212

Nearly optimal-doped high-T_c crystals of cuprate Bi-2212 ($Bi_2Sr_2CaCu_2O_{8+x}$) were used in this work. The critical superconducting temperature T_c in bulk Bi-2212 was verified to be 85 K via transport measurement as shown in Fig S. 1(a). The Bi-2212 intrinsic superconducting gap via our measurements (see details in manuscript on Bi-2212/1T- TaS_2 junctions and supplementary information on Bi-2212/graphite junction below) is around 38-42 meV at 5 K, which reveals the BCS gap ratio $2\Delta_{sc}/k_BT_c$ to be around 10.4-11.5, in good agreement with previous works on the intrinsic Bi-2212 superconducting gap[1, 2].

1T-TaS_2

The 1T-TaS_2 used in our Bi-2212/1T-TaS_2 junctions belongs to the layered transition metal dichalcoginide (TMD) family and has a series of CDW states persisting over a wide temperature range. The thin-flake 1T-TaS_2 transport measurement (shown in Fig. S. 1(b)) reveals with decreasing temperature a transition from a nearly commensurate CDW state (NCCDW) to a commensurate CDW state (CCDW)[3]. The CDW state is a highly ordered configuration in which the central Ta atom is surrounded by 12 Ta atoms forming a "star of David" array[4]) which appears near 180 K on cooling and vanishes near 230 K on warming. As temperature decreases, the resistance increases and transport is dominated by a Mott-CCDW ground state in which the Mott insulating state coexists (or resides in) the CCDW

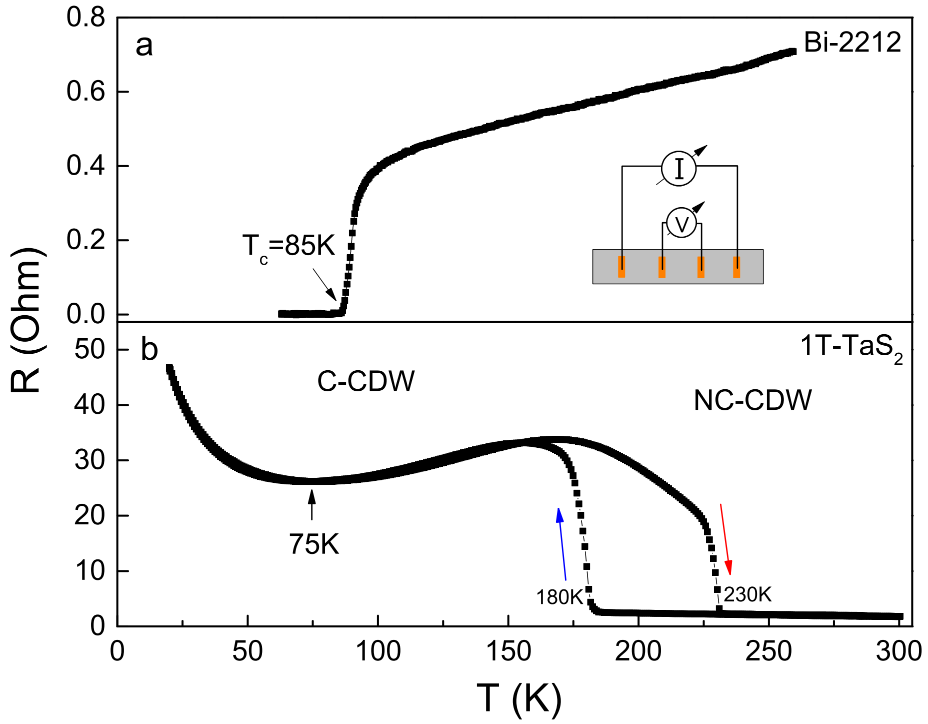

Supplementary Fig S. 1. Intrinsic crystal characteristics. (a): Zero field AC four-terminal transport measurement of Bi-2212 single crystalline flake, indicating the superconducting critical temperature at 85 K. (Inset: Schematic configuration of four-terminal transport measurement.) (b): Zero field AC four-terminal transport measurement of 1T-TaS_2 single crystalline flake. Upward blue and downward red arrows indicate the critical temperatures of CCDW-NCCDW phase transition at cooling and warming process respectively. The vertical arrow indicates the temperature near 75 K where the resistivity begins to increase with decreasing temperature.

state. The upturn in resistivity starting at around 75 K indicates the localization of electrons due to a strong electron-electron Coulomb interaction considered to be the mechanism for the formation of the Mott-insulating state[5–8]. To verify the intrinsic superconducting gap of thin flake Bi-2212 (thickness around 0.5 to 5 μm) along c-axis, we measured several Bi-2212/graphite junctions by replacing the 1T-TaS_2 with graphite and using the same technique as discussed in the manuscript. The freshly exfoliated highly ordered pyrolytic graphite (HOPG) flake is a van der Waals electrode with high conductivity. Consequently, a good normal metal-insulator-superconductor (NIS) or normal metal-superconductor (NS) junction is naturally formed.

BI-2212/GRAPHITE JUNCTION

As shown in Fig. S. 2(a), the differential conductance $(dI/dV)_S$, normalized by the normal state conductance $(dI/dV)_N$ at 100 K, shown in Fig. S. 2(b), clearly shows the commonly accepted c-axis density of states (DOS) of d-wave superconductors without any evidence for a superconducting proximity effect. At low temperatures, the superconducting gap measured from the Bi-2212/graphite junction is around 40 meV. This result not only shows good agreement of the intrinsic gap of Bi-2212 with previous tunneling spectroscopy studies[1, 2], but also reveals consistency with the Bi-2212/1T-TaS_2 junctions discussed in the manuscript. In addition, with a BCS gap function ratio $2\Delta_0/k_BT_c$ =11 and $T_c = 85$ K, we find for our samples the measured temperature dependence of the superconducting gap is well described by the BCS gap function[9] as shown in Fig. S. 2(c).

THEORETICAL MODELING

This section is mainly focused on the theoretical calculation for understanding the density of states (DOS) features at Bi-2212/1T-TaS_2 interface with the effect of superconducting proximity at an energy scale within the intrinsic superconducting gap. The discussion of the multiple dip-hump structures in the manuscript will not be treated in a more quantitative way, since there is no theoretical consensus on the physical mechanisms of peak-dip-hump structures seen in high-T_c cuprates[10–13].

For normal metal-insulator-superconductor (NIS) junctions, the conductance spectrum is calculated using two reflection rates: ordinary reflection (OR) in which incident electrons are reflected as electrons R_{ee} and Andreev reflection (AR) in which incident electrons are reflected R_{eh} as holes. These parameters, R_{ee} and R_{eh}, appear in the Bogoliubov-de Gennes (BdG) equations and are used in the formula of Blonder-Tinkham-Klapwidjk (BTK) on the normal material side of NIS (or NS) junction[14] to describe the current

$$\begin{aligned} I_{NS} &= 2N(0)ev_F A \int_{-\infty}^{+\infty} [f_{\rightarrow}(E) - f_{\leftarrow}(E)]dE \\ &= 2N(0)ev_F A \int_{-\infty}^{+\infty} [f_0(E-eV) - f_0(E)][1 + R_{eh}^2(E) - R_{ee}^2(E)]dE \ , \end{aligned} \tag{S.1}$$

where, A is the area of interface, $f_0(E)$ is the Fermi-Dirac distribution at temperature T, v_F

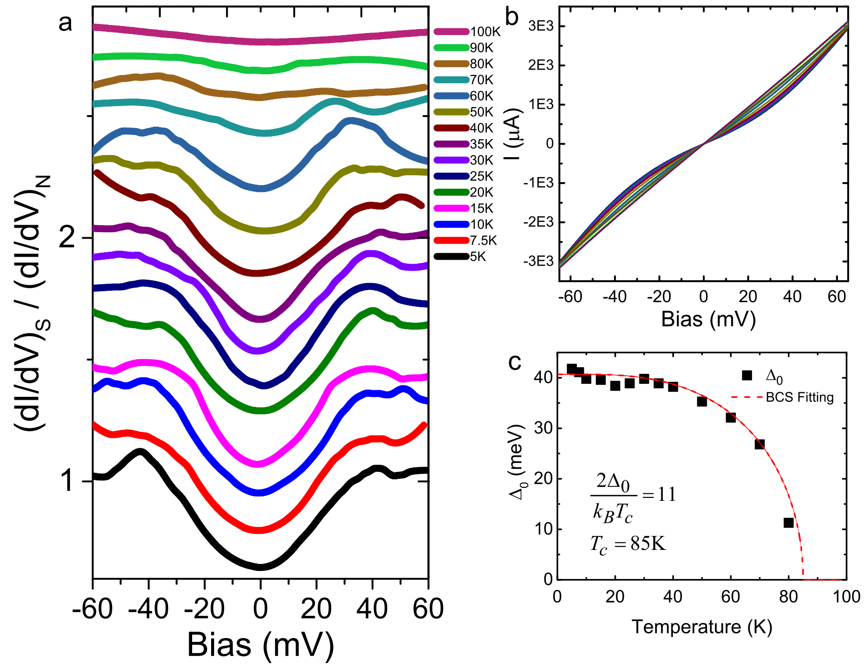

Supplementary Fig S. 2. Bi-2212/graphite junction measurements. (a): The AC differential conductance normalized by the differential conductance at 100 K, $(dI/dV)_S/(dI/dV)_N$, for various temperatures from 5 K to 100 K. The colored isotherms with temperatures identified in the legend of panel (a) are shifted for clarity. (b): DC $I-V$ curves at selected temperatures related to the AC differential conductance measurement shown in panel (a). (c): Temperature dependence of the intrinsic superconducting gap Δ_0 of thin flake Bi-2212, experimentally determined from the tunneling junction with graphite (black squares). The red dashed line indicates the best fit using the BCS gap function with the strong coupling ratio $2\Delta_0/k_BT_c$ =11.

is the fermi velocity, $N(0)$ is the one-spin density of states at $E = E_F$ in the normal state when $N_S(0) = N_N(0)$, $E << E_F$ for junctions, and

$$\begin{aligned} f_{\rightarrow}(E) &= f_0(E-eV) \\ f_{\leftarrow}(E) &= R_{eh}(E)f_0(E+eV) + R_{ee}f_0(E-eV) + [1-R_{eh}(E)-R_{ee}(E)]f_0(E) \ . \end{aligned} \tag{S.2}$$

Accordingly, the differential conductance dI/dV is expressed as

$$\frac{dI}{dV}(V) = 2N(0)ev_FA\int_{-\infty}^{+\infty}\frac{\partial f_0(E-eV)}{\partial(eV)}[1+R_{eh}(E)-R_{ee}(E)]dE \ . \tag{S.3}$$

In more complicated cases instead of an ideal thin junction, there is a superconducting proximity effect in junctions with larger thickness than the superconducting coherence length

because of the thick boundary or interface[15, 16]. The thicknesses of the proximity region of S and N are d_S and d_N respectively. The schemes of superconducting proximity effect are shown in Fig. 4(a) in the manuscript.

However, the original BTK model[14] depicts the tunneling spectrum in the case of conventional superconductors where the superconducting gap is isotropic in momentum space. For unconventional superconductors, with anisotropic gap symmetry in momentum-space, such as d-wave, p-wave, $s+d$ wave, etc, the BTK model needs to be modified. Since Bi-2212 is a well-known high-T$_c$ cuprate superconductor with d-wave gap symmetry in momentum-space[17–21], we start our theoretical calculation based on the previous works of the extended BTK model on tunneling spectrum for d-wave unconventional superconductors [22–24].

The BdG equations for unconventional superconductors with the momentum dependent pairing potential $\Delta(\gamma, r)$ are expressed as

$$\begin{aligned} Eu(\gamma, r) &= H_0(r)u(\gamma, r) + \Delta(\gamma, r)v(\gamma, r) \\ Ev(\gamma, r) &= -H_0(r)v(\gamma, r) + \Delta^*(\gamma, r)u(\gamma, r)\ , \end{aligned} \tag{S.4}$$

where $\gamma = \frac{k}{k_{FS}}$; $u(\gamma, r)$ and $v(\gamma, r)$ are the solutions of the BdG equations for electron-like (ELQ) and hole-like (HLQ) quasiparticles. The Hamiltonian part is $H_0(r) = -\hbar^2\nabla_r^2/2m - \mu + V(r)$.

For c-axis tunneling in d-wave superconductors[22], the ELQ (+) and HLQ (–) are experiencing the same pairing potential magnitude ($|\Delta_+| = |\Delta_-| = \Delta_0\cos(2\alpha)$, α is the angle of one specific orientation away from the lobe of gap in momentum-space), and global phase ($\phi_+ = \phi_- = 0$). So, after solving the BdG equations with boundary conditions[22–24], the AR rate $R_{eh}(E)$ and OR rate $R_{ee}(E)$ at energy E away from E_F are found to be

$$\begin{aligned} R_{eh}(E) &= \frac{e^{-i\theta_+}\sqrt{E+\Omega_-}\sqrt{E-\Omega_+}}{(1+Z^2)\sqrt{E+\Omega_-}\sqrt{E+\Omega_+} - e^{i(\theta_d+\theta_- -\theta_+)}Z^2\sqrt{E-\Omega_-}\sqrt{E-\Omega_+}} \\ R_{ee}(E) &= \frac{-Z(i+Z)[\sqrt{E+\Omega_-}\sqrt{E+\Omega_+} - e^{i(\theta_d+\theta_- -\theta_+)}\sqrt{E-\Omega_-}\sqrt{E-\Omega_+}]}{e^{2iq^+d_N}(1+Z^2)\sqrt{E+\Omega_-}\sqrt{E+\Omega_+} - e^{i(\theta_d+\theta_- -\theta_+)}Z^2\sqrt{E-\Omega_-}\sqrt{E-\Omega_+}}\ . \end{aligned} \tag{S.5}$$

Here, $\Omega_\pm = \sqrt{E^2 - \Delta_0^2}$, $Z = \frac{2mH}{\hbar^2 k_F \cos\theta}$, $q^+ = \sqrt{k_F^2 + 2mE/\hbar^2}\cos\theta$, θ is the angle of the incident orientation of injected electrons relative to the normal orientation of interface, and Z is the well-known BTK parameter representing the dimensionless normal conductance or transparency of interface $\sigma_N = \frac{4\lambda}{(1+\lambda)^2+4Z^2}$, where the parameter λ describes the mismatch ratio of interface $\lambda = k_{FS}/k_{FN}$. For simplicity, we set $k_{FN} = k_{FS}$ resulting in equations. (S.5).

In addition, the parameter θ_d in equations. (S.5) is a parameter corresponding to the thicknesses of the proximity regions. For the case in which only the N side has proximity effect, as proposed in references[22–24], $\theta_d = \frac{4md_N E}{\hbar^2 k_F \cos\theta}$. If we use the BCS coherence length of intrinsic superconductor $\xi_0 = \hbar v_F/\pi\Delta_0$ then $\theta_d = \frac{4}{\pi\cos\theta}\frac{d_N}{\xi_0}\frac{E}{\Delta_0}$[25].

However, generically, since there is a superconducting proximity effect on both sides of S and N[15, 16], we can slightly modify the parameter to be different within three regions, $-d_S < x < 0_-$, $0_- < x < 0_+$ and $0_+ < x < d_N$:

$$\theta_d(x) = \begin{cases} \frac{4}{\pi\cos\theta}\frac{d_N}{\xi_0}\frac{E}{\Delta_0} & 0_+ < x < d_N \\ \frac{4}{\pi\cos\theta}\frac{d_{vdW}}{\xi_0}\frac{E}{\Delta_0} & 0_- < x < 0_+ \\ \frac{4}{\pi\cos\theta}\frac{d_S}{\xi_0}\frac{E}{\Delta_0} & -d_S < x < 0_- \end{cases}, \tag{S.6}$$

where d_{vdW} is the effective thickness of the Van der Waals stacking length.

With the extended tunneling model[22, 23], the normalized differential conductance $\frac{(dI/dV)_S}{(dI/dV)_N}(V)$ is calculated by equation. (S.7) expressed below

$$\frac{(dI/dV)_S}{(dI/dV)_N}(V) = \int_{-\infty}^{+\infty} \frac{\partial f_0(E - eV)}{\partial(eV)} \sigma_T(E) dE \ , \tag{S.7}$$

where $\sigma_T(E) = \frac{\int_\Omega [1 + R_{eh}^2(E) - R_{ee}^2(E)]\sigma_N \cos\theta d\Omega}{\int_\Omega \sigma_N \cos\theta d\Omega}$ and Ω refers to the semi-spherical solid angle integration over the Fermi surface of the d-wave superconductor.

In addition, we consider how the thicknesses of proximity regions could affect the tunneling features, the quasiparticle life time (τ_R) or the scattering rate ($1/\tau_R$). Different quasiparticle scattering rates inside superconducting regions corresponding to the intrinsic and proximate superconducting gaps will have different smearing effects near the edge of the superconducting gap [26]. Following the result by Dynes *et al*, the finite quasiparticle lifetime induced smearing effect on tunneling spectroscopy is calculated via a simple modification by including an imaginary term $-i\Gamma$ in $\sqrt{E \pm \Omega_\pm}$ and $\Omega_\pm$ so that $E \to E - i\Gamma$ and $\Omega_\pm \to \sqrt{(E - i\Gamma)^2 - \Delta_0^2}$. Such a method was also demonstrated by Plecenik *et al.* by including an additional term $-i\Gamma$ in the Hamiltonian of the BdG equations [27]. The term Γ is the quasiparticle lifetime parameter, as $\Gamma = \hbar/\tau_R$[27], and $\frac{1}{\tau_R} = (\frac{k_B T}{\Delta_0})^{1/2}\frac{1}{\tau_0}e^{-\Delta_0/k_B T}$ [26], where τ_0 is a parameter related to the electron-phonon coupling strength. As temperature increases, the quasiparticle scattering rate increases resulting in a broadening of the tunneling spectrum features.

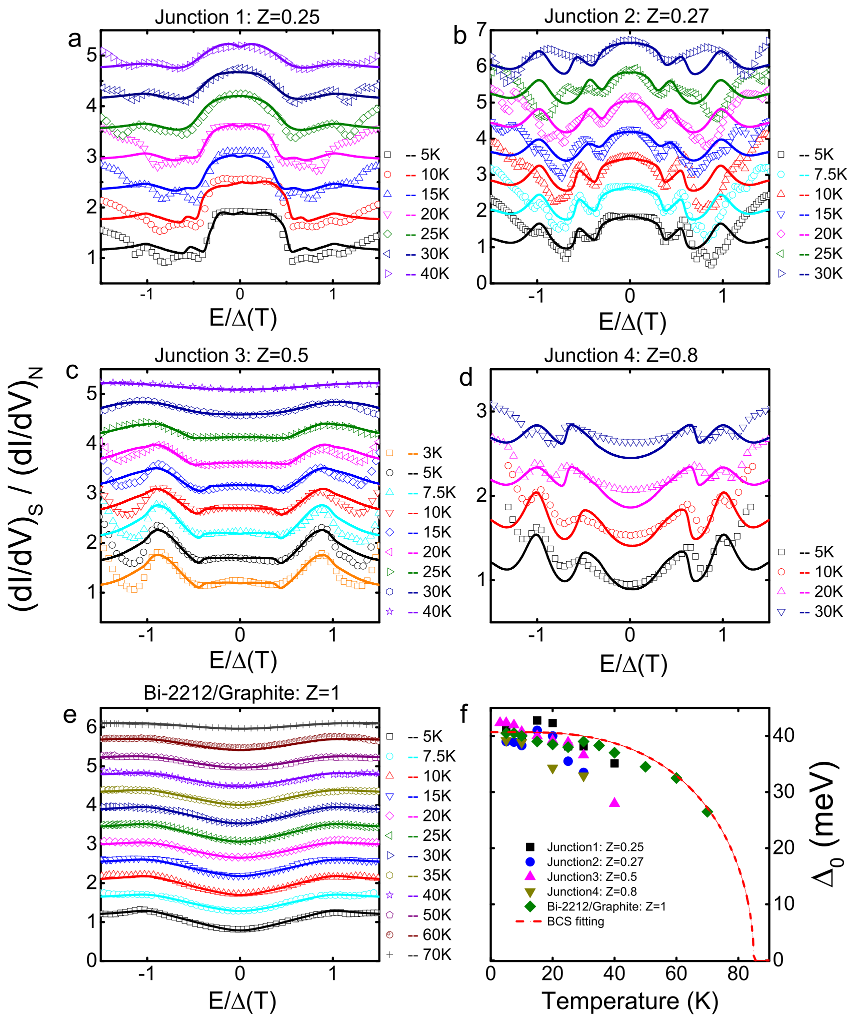

Supplementary Fig S. 3. Calculated normalized conductance $(dI/dV)_S/(dI/dV)_N$ (solid curves) compared to experimental measurement (hollow symbols). (a)–(e): Parameters used in calculations at 5K are listed in the Table I of manuscript. Curves at different temperatures are shifted for clarity. (f): Temperature dependent $\Delta_0^{\mathrm{theory(T)}}$ used in calculation for various junctions. Red dashed line indicates the best fitting by BCS gap function with a ratio $2\Delta_0/k_BT_c$ =11.

The calculated normalized differential conductance $(dI/dV)_S/(dI/dV)_N$ for various junctions, Bi-2212/1T-TaS$_2$ (Z=0.25, 0.27, 0.5 and 0.8) and Bi-2212/Graphite (Z=1), compared to the normalized conductance from measurement are shown in Fig S. 3. The results reveal good agreement between the theoretical model and experimental measurement. Based on the parameters used in theoretical modeling and calculation: Z is the parameter of BTK

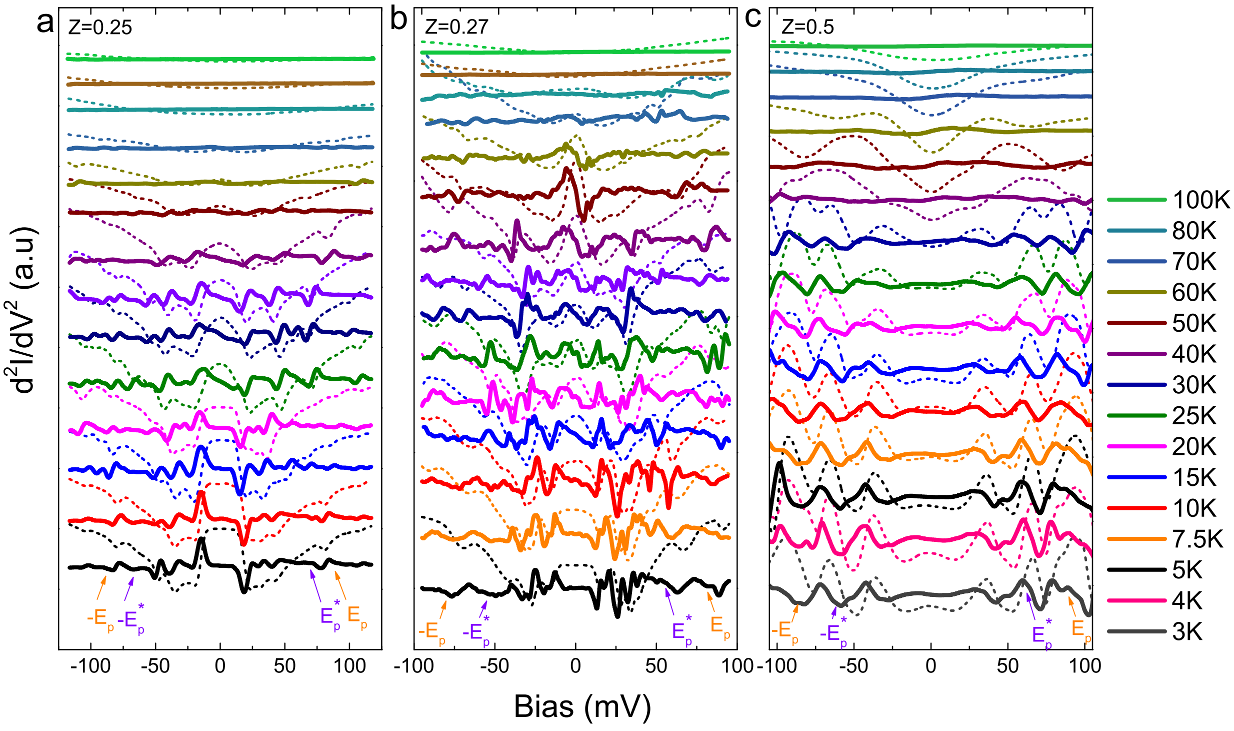

Supplementary Fig S. 4. dI^2/d^2V spectrum of Bi-2212/1T-TaS_2 junctions. Solid lines indicate the curves of dI^2/d^2V spectrum in arbitrary units, and dashed lines indicate the normalized differential conductance $(dI/dV)_S/(dI/dV)_N$. Curves are shifted for clarity. (a): dI^2/d^2V spectrum of Junction-1 (BTK Z=0.25) for various temperatures from 5 K to 100 K. (b): dI^2/d^2V spectrum of Junction-2 (BTK Z=0.27) for various temperatures from 5 K to 100 K. (c): dI^2/d^2V spectrum of Junction-3 (BTK Z=0.5) for various temperatures from 3 K to 100 K. $\mathrm{E_p}$ and $\mathrm{E_p^*}$, indicated by orange and purple arrows, correspond to the peaks position of two dip-hump structures under conductance spectroscopies in dI^2/d^2V spectrum for junctions. At 5K, for Junction-1: $\mathrm{E_p} = 88\,\mathrm{meV}$ and $\mathrm{E_p^*} = 64\,\mathrm{meV}$; for Junction-2: $\mathrm{E_p} = 79\,\mathrm{meV}$ and $\mathrm{E_p^*} = 56\,\mathrm{meV}$; for Junction-3: $\mathrm{E_p} = 89\,\mathrm{meV}$ and $\mathrm{E_p^*} = 62\,\mathrm{meV}$.

model[14]; Γ_0, Γ_p and Γ_a are the quasiparticle lifetime parameters related to Δ_0, Δ_p and Δ_a respectively. The parameters used in the calculation at 5 K for various junctions are listed in Table I in the manuscript.

RECOGNIZING TWO BOSON MODES FROM dI^2/d^2V SPECTRA

As theory in strong-coupling superconductivity describes[28–33], the impact of electron–phonon interactions on the density of states of superconductors is predicted to occur near energies $E = \Delta + \Omega$, where Δ is the superconducting gap and Ω is the corresponding boson mode energy. Such signature corresponding to the electron–phonon interaction is the dip-hump structure in the conductance spectroscopies (dI/dV vs. V) of NIS junctions. The boson mode energy Ω is obtained by identifying the signature of a peak in the positive bias region or a dip in the negative bias region at an energy scale larger than superconducting gap in the d^2I/dV^2 spectra. Also, the peak/dip feature in d^2I/dV^2 spectra corresponds to the position where the dip-hump structure in conductance spectroscopy dI/dV has the maximum magnitude of slope. The energy scales of the two boson modes arising from the double dip-hump structures discussed in the manuscript for the Bi-2212/1T-TaS_2 Junctions 1–3 could be obtained by numerically calculating the first order derivative of conductance spectroscopy dI/dV. A small amount of deviation is foreseeable since the conductance spectroscopy data are acquired in incremental steps of 1 mV. We argue that these deviations do not obscure the features of the double dip-hump structures. The d^2I/dV^2 spectra of Junctions 1–3 at various temperatures up to 100 K are shown in Fig S. 4. Two dips in the negative bias region and two peaks in the positive bias region are marked by orange and purple arrows respectively, which are related to the maximum slopes of the two dip-hump structures in conductance spectroscopies at energy E_p and E_p^*, as indicated by the dashed lines.

Bi-2212 is a d–wave superconductor, the c-axis tunneling spectroscopies incorporate and average all possible gaps along momentum space. There is no consensus on the pairing mechanism of superconductivity in high-T_c cuprates, and the consistency of the electron-boson interaction on nodal and anti-nodal regimes in momentum space is still unknown. Nonetheless, the good agreement of Bi-2212 intrinsic superconducting gap measured from Bi-2212/1T-TaS_2 and Bi-2212/graphite junctions with previous results on intrinsic Bi-2212 single crystal as discussed above and in the manuscript probably reveals the superconducting gap varies monotonically from the nodal to the anti-nodal regime along momentum space, which could clarify the rationality of the method for obtaining the boson mode energies from c-axis conductance spectroscopies and dI^2/dV^2.

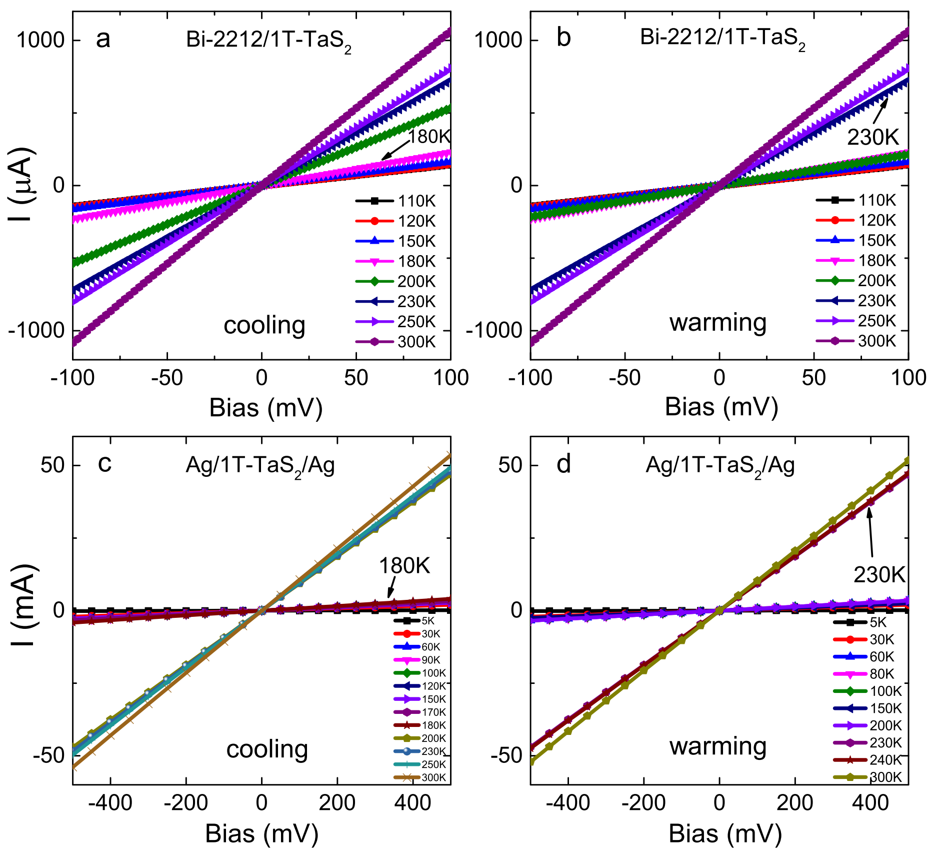

Supplementary Fig S. 5. $I-V$ characteristics of (a)–(b) Bi-2212/1T-TaS_2 Junction-1 (Z=0.25) at temperatures above Bi-2212's T_c and (c)–(d) back-to-back structured Ag/1T-TaS_2/Ag junction for cooling and warming processes.

$I-V$ CHARACTERISTICS OF BI-2212/1T-TAS_2 (ABOVE T_c) AND AG/1T-TAS_2/AG JUNCTIONS

DC $I-V$ characteristics of Bi-2212/1T-TaS_2 Junction-1 (Z=0.25) at temperatures above Bi-2212's T_c and back-to-back structured Ag/1T-TaS_2/Ag junction under one cooling/warming cycle are shown in Fig. S. 5. The Ag/1T-TaS_2/Ag junction are constructed by SPI Ag paste on 1T-TaS_2.

Supplementary References

[1] Renner, Ch. *et al.* Observation of the low temperature pseudogap in the Vortex Cores of $Bi_2Sr_2Ca_1Cu_2O_{8+\delta}$. *Phys. Rev. Lett.* **80**, 3606–3609 (1998).

[2] Renner, Ch. & Fischer, Ø. Vacuum tunneling spectroscopy and asymmetric density of states of $Bi_2Sr_2Ca_1Cu_2O_{8+\delta}$. *Phys. Rev. B* **51**, 9208–9218 (1995).

[3] Di Salvo, F. J. & Graebner, J. E. The low temperature electrical properties of 1T-TaS_2. *Solid State Commun.* **22**, 825–828 (1977).

[4] Wilson, J. A. & Di Salvo, F. J. & Mahajan, S. Charge-density waves and superlattices in the metallic layered transition metal dichalcogenides. *Adv. Phys.* **24**, 117–201 (1975).

[5] Fazekas, P. in *Modern Trends in the Theory of Condensed Matter: Lecture Notes in Physics* vol. 115, 328–338 (Springer, 1980).

[6] Fazekas, P. & Tosatti, E. Charge carrier localization in pure and doped 1T-TaS_2. *Physica B+C* **99**, 183187 (1980).

[7] Thomson, R. E., Burk, B., Zettl, A. & Clarke, J. Scanning tunneling microscopy of the charge-density-wave structure in 1T-TaS_2. *Phys. Rev. B* **49**, 16899–16916 (1994).

[8] Kim, J. J., Yamaguchi, W., Hasegawa, T. & Kitazawa, K. Observation of Mott localization gap using low temperature scanning tunneling spectroscopy in commensurate 1T-TaS_2. *Phys. Rev. Lett.* **73**, 2103–2106 (1994).

[9] Tinkham, M., *Introduction to Superconductivity.* Dover Publications (1996).

[10] Eschrig, M. & Norman, N. R. Neutron Resonance: Modeling photoemission and tunneling data in the superconducting state of $Bi_2Sr_2Ca_1Cu_2O_{8+\delta}$. *Phys. Rev. Lett.* **85**, 3261–3264 (2000).

[11] Alexandrov, A. S. & Sricheewin, C. Theory of SIS tunneling in the cuprates. *Europhys. Lett.* **58**, 576–581 (2002).

[12] Gabovich, A. M. & Voitenko, A. I. Charge–density–wave origin of the dip–hump structure in tunnel spectra of the BSCCO superconductor. *Phys. Rev. B* **75**, 064516 (2007).

[13] Chen, J-W., Kao, Y-J. & Wen, W-Y. Peak–dip–hump lineshape from holographic superconductivity. *Phys. Rev. D* **82**, 026007 (2010).

[14] Blonder, G. E., Tinkham, M. & Klapwidjk, T. M. Transition from metallic to tunneling regimes in superconducting microconstrictions: Excess current, charge imbalance, and supercurrent

conversion. *Phys. Rev. B* **25**, 4515–4532 (1982).

[15] De Gennes, P. G. Boundary effects in superconductors. *Rev. Mod. Phys.* **36**, 225–237 (1964).

[16] McMillan, W. L. Tunneling model of the superconducting proximity effect. *Phys. Rev.* **175**, 537–542 (1968).

[17] Dessau, D. S. *et al.* Key features in the measured band-structure of $Bi_2Sr_2Ca_1Cu_2O_{8+\delta}$ at bands at ε_F and fermi-surface nesting. *Phys. Rev. Lett.* **71**, 2781–2784 (1993).

[18] Shen, Z. X., Spicer, W. E., King, D. M, Dessau, D. S., & Wells, B. O. Photoemission Studies of High-Tc Superconductors: The Superconducting Gap. *Science* **267**, 5296 (1995).

[19] Ding, H. *et al.* Angle-resolved photoemission spectroscopy study of the superconducting gap anisotropy in $Bi_2Sr_2Ca_1Cu_2O_{8+\delta}$. *Phys. Rev. B* **54**, R9678–R9681 (1996).

[20] Norman, M. R. *et al.* Destruction of the Fermi surface underdoped high-Tc superconductors. *Nature* **392**, 157160 (1998).

[21] Valla, T. *et al.* Evidence for quantum critical behavior in the optimally doped cuprate $Bi_2Sr_2Ca_1Cu_2O_{8+\delta}$. *Science* **285**, 2110–2113 (1999).

[22] Kashiwaya, S., Tanaka, Y., Koyanagi, M., Takashima, H. & Kajimura, K. Origin of zero-bias conductance peaks in high-T_c superconductors. *Phys. Rev. B* **51**, 1350–1353 (1995).

[23] Kashiwaya, S., Tanaka, Y., Koyanagi, M. & Kajimura, K. Theory for tunneling spectroscopy of anisotropic superconductors. *Phys. Rev. B* **53**, 2667–2676 (1996).

[24] Kashiwaya, S. & Tanaka, Y. Tunneling effects on surface bound states in unconventional superconductors. *Rep. Prog. Phys.* **63**, 1641–1724 (2000).

[25] Annett, J. in *Superconductivity, Superfluids and Condensates.*, 62-63 (New York: Oxford university press, 2004).

[26] Dynes, R. C., Narayanamurti, V. & Garno, J. P. Direct measurement of quasiparticle-lifetime broadening in a strong-coupled superconductor. *Phys. Rev. Lett.* **41**, 1509–1511 (1978).

[27] Plecenik, A., Grajcar, M., Beňačka, Š., Seidel, P. & Pfuch, A. Finite quasiparticle lifetime effects in the differential conductance of $Bi_2Sr_2Ca_1Cu_2O_{8+\delta}$/Au junctions. *Phys. Rev. B* **49**, 10016–10019 (1994).

[28] McMillan, W. L. & Rowell, J. M. "Superconductivity". **1** (ed. Parks, R. D.) 561 (Dekker, New York, 1969).

[29] Scalapino, D. J. "Superconductivity". **1** (ed. Parks, R. D.) 561 (Dekker, New York, 1969).

[30] Carbotte, J. P. Properties of boson-exchange superconductors. *Rev. Mod. Phys.*, **62**, 1027–1157 (1990).

[31] Eliashberg, G. M. Interactions between electrons and lattice vibrations in a superconductor. *Sov. Phys. JETP*, **11**, 696–702 (1960).

[32] Wolf, E. L. “Principles of Electron Tunneling Spectroscopy”, Oxford University Press, New York (1989) Chapter 4.

[33] Gabovich, A. M. & Voitenko, A. I. Charge-density-wave origin of the dip-hump structure in tunneling spectra of the BSCCO superconductor. *Phys. Rev. B*, **75**, 064516 (2007).
